# Muscular dystrophy–associated lamin variants disrupt cellular organization through a nucleolar-ribosomal axis

**DOI:** 10.1126/sciadv.aec9028

**Published:** 2026-08-26

**Authors:** Xiangyi Ding, Sweta Kumari, Ellen F. Gregory, Daniel A. Starr, G. W. Gant Luxton

**Affiliations:** Department of Molecular and Cellular Biology, University of California, Davis, Davis, CA, USA.

## Abstract

Emery-Dreifuss muscular dystrophy (EDMD) arises from mutations in nuclear lamins or emerin. Current pathological models emphasize defective nuclear mechanics and transcriptional regulation, yet these mechanisms cannot explain how lamina defects propagate across the cell to produce the complex pathology of laminopathies. Here, we reveal an emerging pathway linking nuclear lamina dysfunction to cytoplasmic reorganization. Using *Caenorhabditis elegans* EDMD models, we show that disease-linked lamin variants reduce cytoplasmic mesoscale crowding, increase molecular diffusivity, and disrupt nuclear positioning and endoplasmic reticulum architecture, which mirror phenotypes caused by ribosome depletion. Lamin dysfunction also lowers nucleolar fibrillarin levels and ribosome abundance, revealing a nucleolar-ribosomal axis that transmits nuclear defects to the cytoplasm. Loss of the redundant LEM-domain proteins *emr-1* and *lem-2* phenocopied lamin mutants, indicating that cytoplasmic disorganization is a shared hallmark of EDMD. These findings connect nuclear architecture to whole-cell biophysics and suggest therapeutic strategies aimed at restoring ribosome function.

## INTRODUCTION

Emery-Dreifuss muscular dystrophy (EDMD) is a devastating neuromuscular disorder characterized by progressive skeletal muscle weakness, cardiac conduction defects, and joint contractures (*1*). EDMD arises primarily from mutations in either *LMNA*, encoding nuclear A-type lamins (lamins A/C), or *EMD*, encoding the inner nuclear membrane protein emerin (*1*). A-type lamins confer mechanical stability to the nucleus, connect the nucleus to the cytoskeleton via linker of nucleoskeleton and cytoskeleton (LINC) complexes, and regulate chromatin organization to control gene expression (*2*). Emerin is an inner nuclear membrane protein and member of the LEM-domain family (named for LAP2, Emerin, and MAN1) that interacts with lamins and chromatin to organize the nuclear periphery (*3*, *4*). Together, these observations highlight that EDMD arises from disruption of the lamina network rather than a single protein, underscoring the challenge of understanding how lamina dysfunction mechanistically leads to the tissue-specific EDMD pathologies.

A defining cellular hallmark of EDMD is aberrant nuclear positioning in skeletal muscle fibers (*5*, *6*). In healthy muscle, nuclei distribute peripherally near the sarcolemma, but patients with EDMD show nuclei inappropriately clustered at the fiber center (*5*). This nuclear mispositioning indicates disrupted nuclear-cytoskeletal coupling, likely through LINC complexes (*7*, *8*). However, nuclear positioning defects alone cannot fully explain EDMD’s complex pathophysiology, as other diseases with prominent nuclear mispositioning present distinct progression patterns (*9*), indicating that additional cellular mechanisms must be disrupted.

Current laminopathy models focus primarily on altered mechanotransduction and dysregulated gene expression (*10*). While these mechanisms contribute to disease, neither fully accounts for the broad cellular disorganization observed in EDMD tissues, including ruffled nuclear envelope and mispositioned muscle nuclei (*10*). Recent advances in understanding cytoplasmic biophysical properties suggest a third possibility: lamin mutations might disrupt fundamental physical properties of the cellular interior, leading to widespread organizational defects (*11*, *12*).

The eukaryotic cytoplasm is a highly organized, crowded environment where macromolecular interactions influence all cellular functions (*13*). Macromolecular crowding affects protein folding, enzyme kinetics, liquid-liquid phase separation, and organelle positioning (*13*). Ribosome concentration is a major determinant of cytoplasmic macromolecular crowding and cellular organization in yeast and mammalian cells (*11*). Ribosome depletion reduces cytoplasmic macromolecular crowding, disrupts nuclear positioning, and causes endoplasmic reticulum (ER) network collapse in *C. elegans* (*12*). Ribosomes assemble in the nucleolus before cytoplasmic export, creating a direct link between nuclear function and cytoplasmic organization (*11*, *14*).

*C. elegans* provides unique advantages for investigating potential interactions between nuclear lamins, nucleolar function, and cytoplasmic macromolecular crowding. The nematode has a single lamin gene, *lmn-1*, whose product functionally substitutes for both A- and B-type mammalian lamins (*7*, *15*, *16*). Like human lamins, LMN-1 localizes to the nuclear periphery, interacts with chromatin, and is required for proper nuclear shape and positioning (*7*, *16*, *17*). *C. elegans* EDMD models engineered using CRISPR-Cas9 faithfully recapitulate many disease phenotypes, including reduced fitness, movement disorders, and nuclear morphology defects (*17*). The *lmn-1(R64P)* mutation, corresponding to the severe human variant *LMNA* p.R50P, is particularly disruptive, causing both polymerization defects in vitro, and EDMD-like phenotypes in vivo (*15*, *17*). *C. elegans* is also well suited for measuring macromolecular crowding through in vivo nanorheology using genetically encoded multimeric nanoparticles (GEMs) (*11*, *12*), fluorescent protein assemblies that report local crowding conditions through their diffusive behavior in live animals. This approach revealed that cytoplasmic mesoscale macromolecular crowding in *C. elegans* tissues is regulated by the giant ER/outer nuclear membrane Klarsicht/ANC-1/SYNE homology (KASH) protein ANC-1 and ribosome concentration (*12*), both of which independently influence nuclear positioning and ER morphology.

Here, we test whether EDMD-associated lamin mutations disrupt cellular organization through effects on mesoscale macromolecular crowding, ribosome biogenesis, and nucleolar function. Our findings reveal a nucleolar-ribosomal axis through which nuclear lamina defects propagate to cause broad cellular dysfunction, providing a mechanistic basis for the cellular disorganization observed in EDMD tissues.

## RESULTS

### EDMD-associated lamin variants disrupt cytoplasmic biophysical properties in vivo

**Fig. 1.**
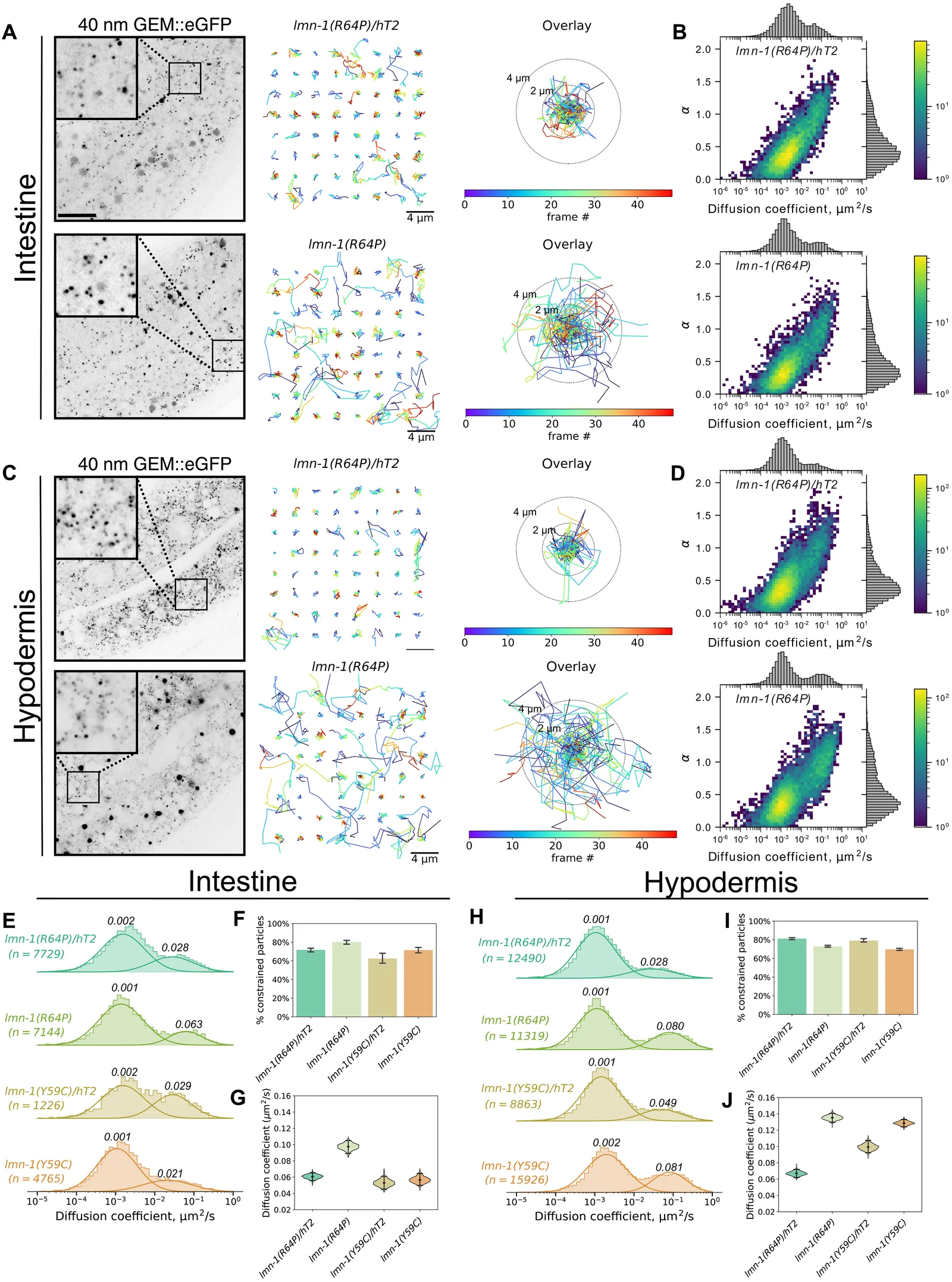
*lmn-1* variants Y59C and R64P disrupt cytoplasmic biophysical properties. (**A** and **C**) Representative inverted grayscale spinning disc confocal images of GEMs in the intestine (A) or hypodermis (C) of day 1 *lmn-1(R64P)/hT2* (top) or *lmn-1(R64P)* (bottom) adults. Scale bar, 10 μm. Insets show magnified views of boxed regions. Right panels display representative GEM trajectories color-coded by frame number. Scale bar, 4 μm. Concentric circles indicate 2 and 4 μm radii from the trajectory origin. (**B** and **D**) Two-dimensional probability density histograms of *D*_eff_ versus the anomalous diffusion exponent (α) over the initial 100 ms for GEM diffusion in the intestine (B) or hypodermis (D) of day 1 *lmn-1(R64P)/hT2* (top) or *lmn-1(R64P)* (bottom) adults. Marginal distributions shown as frequency histograms on top and right axes. Color scale indicates particle count per bin. (**E** and **H**) Gaussian mixture model (GMM) analysis of GEM *D*_eff_ in the intestine (E) or hypodermis (H) for the indicated genotypes. Distributions are plotted in log space; annotated values indicate the geometric mean of each identified peak. (**F**, **G**, **I**, and **J**) Bootstrap analysis (1000 iterations) of GMM-derived GEM diffusion parameters in the intestine (F and G) or hypodermis (I and J). Bar plots (F and I) show the percentage of the constrained GEM population; violin plots (G and J) show the *D*_eff_ of the unconstrained GEM population. All values represent mean ± 95% confidence interval (CI).

To investigate how lamin mutations affect cytoplasmic biophysical properties in living tissues, we used in vivo nanorheology in living *C. elegans*. This approach provides real-time quantification of cytoplasmic biophysical properties in intact animals using 40-nm GEMs as fluorescent tracers to explore the mesoscale intracellular environment (*11*, *12*). We quantified cytoplasmic biophysical properties by analyzing mean squared displacement (MSD) curves to calculate effective particle diffusion coefficients (*D*_eff_) through tracking trajectories of thousands of individual GEMs with high-speed spinning-disc confocal microscopy. Measurements were made in the hypodermis and intestine of homozygous *lmn-1* mutant animals and compared to heterozygous controls maintained by *hT2* balancer (movies S1 and S2) (*17*, *18*). In heterozygous control animals, most GEMs were highly constrained within the crowded cytoplasm, while a smaller population of GEMs showed faster, more diffusive movements (Fig. 1, A and C). This bimodal pattern was consistent with previous observations in wild-type animals and reflects the spatially heterogeneous nature of the intracellular environment (*12*).

We next characterized GEM behavior in EDMD-associated *lmn-1* homozygous mutants, focusing on two early-onset variants: *lmn-1(R64P)* and *lmn-1(Y59C)*, respectively, corresponding to human *LMNA* p.R50P and p.Y45C (*15*, *17*, *19*). The *lmn-1(R64P)* mutation causes polymerization defects in vitro (*15*). Homozygous *lmn-1(R64P)* mutants showed substantial and reproducible increases in GEM mobility compared to balanced heterozygous controls in both the intestine and the hypodermis. In contrast, homozygous *lmn-1(Y59C)* mutants displayed marked increases in GEM mobility in the hypodermis but minimal effects in the intestine (Fig. 1, A and C, and fig. S1). To quantitatively analyze these distinct kinetic populations, we performed MSD analysis (fig. S2) to extract an effective diffusion coefficient (*D*_eff_) and an anomalous exponent (α) for each GEM trajectory (Fig. 1, B and D), and then applied Gaussian mixture modeling to objectively separate trajectories into low and high mobility population using our previously developed analytical framework (Fig. 1, E and H) (*12*). In the diffusivity distributions, the left peak corresponds to GEMs whose motion is frequently hindered by cellular structures (“constrained” GEMs), while the right peak corresponds to GEMs diffusing more freely through the cytoplasm (“unconstrained” GEMs). We indicate the geometric mean of each peak to assist comparison of distribution shifts (Fig. 1, E and H). Homozygous *lmn-1(R64P)* and *lmn-1(Y59C)* mutants both showed a rightward shift in the unconstrained peak relative to their balanced controls, indicating enhanced diffusion of freely moving particles. To rigorously quantify this, we isolated the unconstrained population and used bootstrap analysis (1000 iterations) to estimate the arithmetic mean *D*_eff_. This revealed that *lmn-1(R64P)* markedly increased unconstrained *D*_eff_ in both the intestine and hypodermis (Fig. 1, G and J), whereas *lmn-1(Y59C)* produced pronounced increases only in the hypodermis (Fig. 1, G and J, and fig. S1). Notably, the relative abundance and diffusivity of the constrained fraction remained largely unchanged across all genotypes (Fig. 1, F and I), indicating that lamin dysfunction primarily disrupts mesoscale macromolecular crowding rather than the structural constraints that physically confine GEM movement. Together, these results demonstrate that EDMD-associated lamin mutations alter cytoplasmic biophysical properties by reducing mesoscale macromolecular crowding, albeit with distinct tissue sensitivities.

### EDMD-associated lamin variants recapitulate disease-relevant cellular defects

Disrupted nuclear positioning is consistently observed in the skeletal muscle of patients with EDMD (*1*, *5*). We therefore examined whether our *C. elegans* lamin mutants recapitulate this phenotype by analyzing nuclear positioning in the hypodermis, a single-cell syncytial layer containing 139 nuclei that are evenly spaced apart and anchored in place by mechanisms involving the giant KASH protein ANC-1 (*20*, *21*). We quantified nuclear anchorage defects by measuring the percentage of nuclei found in abnormal clusters (*22*, *23*).

**Fig. 2.**
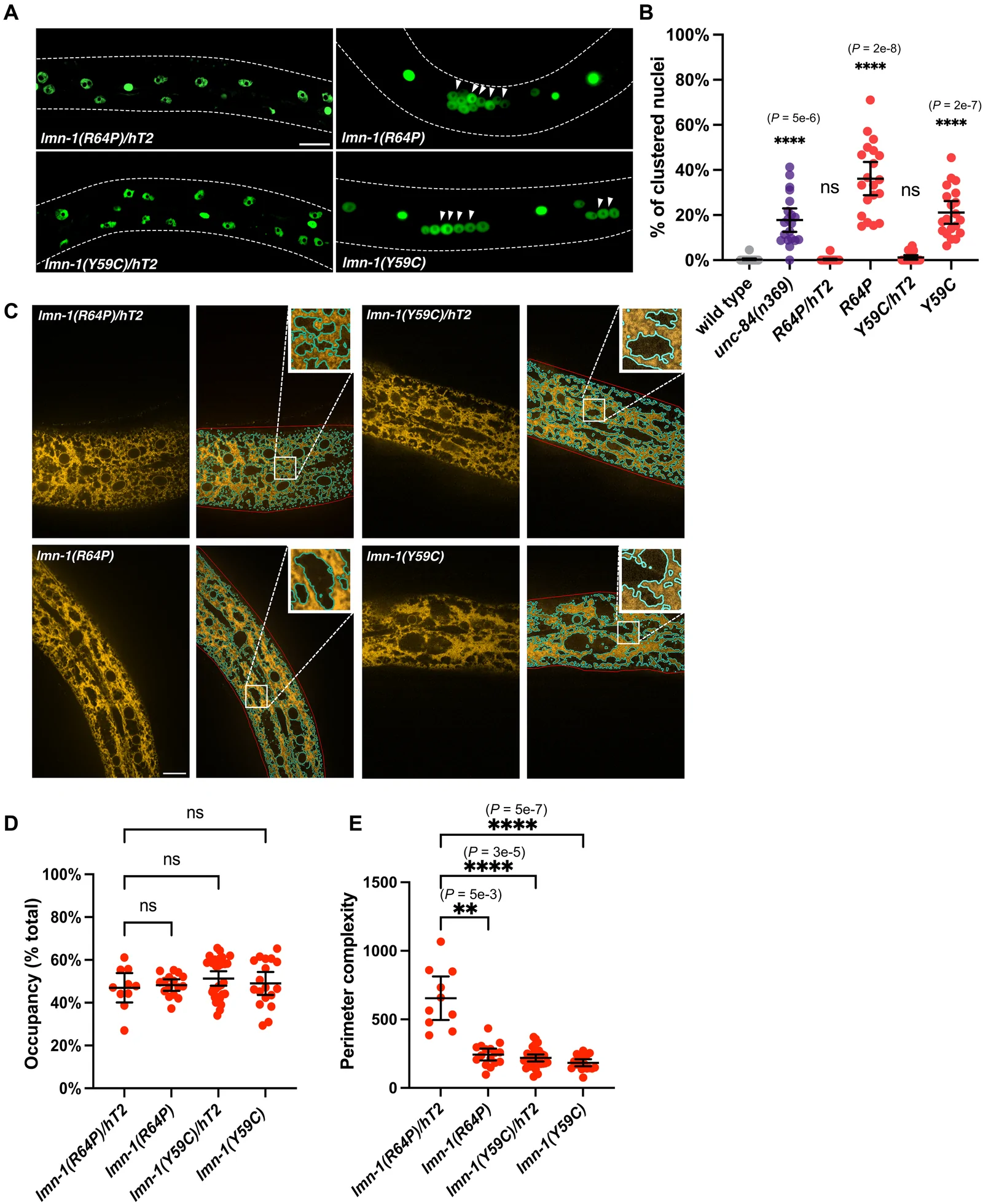
*lmn-1* variants disrupt nuclear positioning and ER morphology in the hypodermis. (**A**) Representative spinning disc confocal (left) or epifluorescence (right) images of GFP-labeled hypodermal nuclei (*ycIs10*, see Materials and Methods) in day 1 adult worms of the indicated strains. The GFP marker labels both hyp7 and seam cell nuclei where seam cell nuclei appear brighter. Only hyp7 nuclei were scored for clustering. Arrowheads indicate clustered hypodermal nuclei; dotted lines outline worm boundaries. Scale bar, 10 μm. (**B**) Nuclear clustering quantified as the percentage of clustered nuclei per worm in day 1 adults of the indicated strains. Each point represents one worm (*n* = 20 for each genotype). Welch’s ANOVA test: *W* (5, 50) = 44, *P* < 0.001. (**C**) Representative spinning disc confocal images of hypodermal ER organization in day 1 adults of the indicated strains. Left panels show ER morphology visualized by mKate2::TRAM-1 fluorescence; right panels show thresholded images with ER outline (cyan) and hypodermal boundary (red). Insets show magnified views of boxed regions. Scale bar, 10 μm. (**D**) ER occupancy (% of total hypodermal area) across the indicated genotypes (*n* = 10, 16, 31, and 18, left to right). Kruskal-Wallis test: for (D), *H* (3) = 1.491, *P* > 0.05 (ns). (**E**) ER perimeter complexity (see Methods) across the indicated genotypes (*n* = 10, 16, 31, and 18, left to right). Kruskal-Wallis test: for (E), *H* (3) = 30.458, *P* < 0.001. Each point represents one worm. Statistical significance is indicated as follows: ****P < 0.0001, ***P ≤ 0.001, **P ≤ 0.01, *P ≤ 0.05, and ns: *P* > 0.05; exact *P* values are shown above each comparison in the figure.

EDMD-associated lamin mutations caused significant nuclear clustering compared to controls (Fig. 2, A and B). The severe *lmn-1(R64P)* variant produced the strongest effect, with ∼35% of nuclei clustered, versus <5% in wild-type animals. *lmn-1(Y59C)* had an intermediate effect, consistent with its less severe impact on cytoplasmic crowding in the hypodermis. This nuclear mispositioning defect parallels those observed in EDMD patient tissues. The severity hierarchy, with R64P showing a stronger defect than Y59C, also matches known in vitro polymerization defects and organismal phenotypes (*15*, *17*), demonstrating that EDMD-associated lamin mutations disrupt the mechanisms governing nuclear positioning.

To examine whether lamin mutations affect broader cellular architecture beyond nuclear positioning, we examined ER morphology. The ER forms extensive networks throughout hypodermal syncytia that depend on *anc-1* pathways involved in cytoplasmic constraint and macromolecular crowding pathways reliant on ribosome concentration (*12*, *20*). In balanced heterozygous controls, the hypodermal ER displays a complex branched network of interconnected tubules and sheets with small spaces between ER-rich regions (Fig. 2C). *lmn-1(R64P)* mutants, however, showed altered ER architecture, with larger void spaces suggesting network collapse (Fig. 2C). The mutant ER formed more sheet-like structures with reduced tubular branching, resembling simplified networks observed under cytoplasmic stress or crowding defect (*24*). We quantified these defects using described morphometric approaches (*12*, *25*), measuring both ER occupancy (total area coverage) and network complexity (perimeter-to-area ratio). While *lmn-1(R64P)* mutants showed no significant change in total ER area, they exhibited marked reduction in network complexity (Fig. 2, D and E). Intriguingly, the *lmn-1(Y59C)* variant displayed a distinct pattern for this morphological phenotype; both *lmn-1(Y59C)/hT2* heterozygotes and *lmn-1(Y59C)* homozygotes exhibited significantly reduced perimeter complexity (Fig. 2E). This finding suggests that the Y59C mutation exerts a dominant effect on ER network architecture that is independent of its effects on bulk cytoplasmic crowding, distinguishing it mechanistically from *lmn-1(R64P)*. Notably, the nature of ER network defects in *lmn-1(R64P)* mutants closely matched those previously observed in ribosome-depleted animals (*12*), suggesting a shared underlying mechanism. These data demonstrate that lamin dysfunction produces cellular defects quantitatively and qualitatively similar to ribosome depletion effects, pointing toward a common pathway linking nuclear lamina integrity to cytoplasmic organization.

### LMN-1 and ribosomes function in a common pathway controlling cellular organization

Since *lmn-1(R64P)* mutants phenocopy several cellular organization defects previously observed in ribosome-depleted animals, including reduced cytoplasmic crowding, nuclear mispositioning, and ER network collapse, we hypothesized that lamins and ribosomes function in the same pathway to regulate mesoscale macromolecular crowding and cellular organization. This model predicts that if lamins act upstream of ribosomes in the same pathway, combining *lmn-1* mutations with partial ribosome depletion should produce epistatic rather than additive effects. To test this prediction, we performed genetic interaction studies using *rps-18(RNAi)* to partially deplete ribosomes in various *lmn-1* mutant backgrounds and quantified three independent phenotypes: GEM diffusion, nuclear positioning, and ER architecture.

**Fig. 3.**
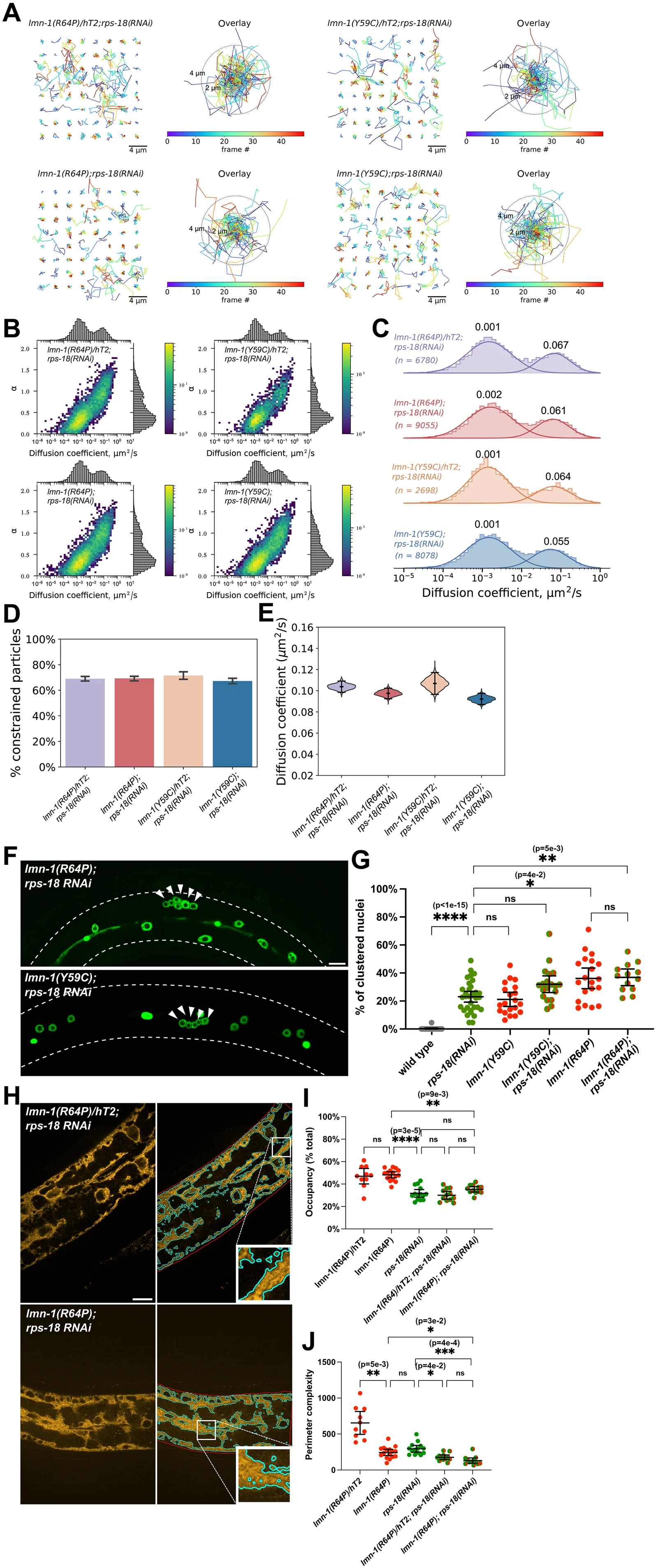
LMN-1 and ribosomes act in the same pathway to control cytoplasmic organization. (**A**) GEM trajectories color-coded by frame number in intestinal cytoplasm of the indicated strains. Scale bar, 4 μm. Concentric circles indicate 2 and 4 μm radii from the trajectory origin. (**B**) Two-dimensional probability density histograms of GEM *D*_eff_ versus α. Marginal histograms are shown on top and right axes. Color scale indicates particle count per bin. (**C**) GMM analysis of intestinal GEM *D*_eff_. (**D** and **E**) Bootstrap analysis (1000 iterations) of GMM-derived intestinal GEM diffusion parameters. Bar plots (D) show the percentage of the constrained GEM population; violin plots (E) show *D*_eff_ of the unconstrained population. All values represent mean ± 95% CI. (**F**) Representative spinning disc confocal images of GFP-labeled hypodermal nuclei. The marker labels both hyp7 and seam nuclei, with seam nuclei appearing brighter. Only hyp7 nuclei were scored. Arrowheads indicate clustered nuclei; dotted lines outline worm boundaries. Scale bar, 10 μm. (**G**) Nuclear clustering quantified as the percentage of clustered nuclei per worm (*n* = 20, 33, 20, 19, 20, and 13, left to right). Welch’s ANOVA: *W* (5, 43) = 115, *P* < 0.001. (**H**) Representative images of hypodermal ER organization. Left: mKate2::TRAM-1 fluorescence; right: thresholded images with ER outline (cyan) and hypodermal boundary (red). Insets show magnified boxed regions. Scale bar, 10 μm. (**I** and **J**) ER occupancy (I) and perimeter complexity (J; see Materials and Methods) across the indicated genotypes (*n* = 10, 16, 14, 12, and 12, left to right). Kruskal-Wallis: *H* (4) = 40 for (I) and 44 for (J), *P* < 0.001. Each point represents one worm. Significance: ****P < 0.0001, ***P ≤ 0.001, **P ≤ 0.01, *P ≤ 0.05, and ns: *P* > 0.05; exact *P* values are shown above each comparison in the figure.

Ribosome depletion by *rps-18(RNAi)* robustly increased intestinal GEM diffusion in heterozygous *lmn-1(R64P)/hT2*, *lmn-1(Y59C)/hT2*, and homozygous *lmn-1(Y59C)* animals, as shown by GEM trajectories, two-dimensional probability density histograms, and quantitative analyses (Figs. 1, E and G; and 3, A to C and E), reproducing previous findings that ribosomes regulate cytoplasmic crowding (*12*). This response demonstrated that ribosome depletion retained its ability to disrupt cytoplasmic organization in genetic backgrounds with partial lamin function. However, *rps-18(RNAi)* failed to further increase cytoplasmic GEM diffusion in homozygous *lmn-1(R64P)* mutants, revealing a clear epistatic interaction (Figs. 1G and 3, B to E). This epistasis suggests that the severe *lmn-1(R64P)* mutation maximally disrupts the lamin-ribosome pathway. The differential responses between *lmn-1(Y59C)* (additive effects) and *lmn-1(R64P)* (epistatic effects) provide insight into the severity hierarchy of EDMD variants and their relative impacts on the lamin-ribosome regulatory axis.

Nuclear positioning assays revealed similar epistatic patterns. While *rps-18(RNAi)* slightly enhanced nuclear clustering defects in *lmn-1(Y59C)* mutants, it produced no additional defect in *lmn-1(R64P)* backgrounds (Fig. 3, F and G). This consistent pattern across two independent cellular phenotypes strengthened the evidence for pathway convergence and indicated that genetic interactions were not specific to cytoplasmic crowding effects alone. As predicted by the pathway model, the ER morphology parameters in *rps-18(RNAi); lmn-1(R64P)* double-mutant animals remained comparable to *rps-18(RNAi)* heterozygous controls (Fig. 3, H to J), completing the epistatic pattern across all three cellular organization phenotypes we examined. Thus, across all three phenotypes, LMN-1 and ribosomes act in a common pathway controlling cellular organization.

### LMN-1 regulates the nucleolar-ribosomal axis

Having demonstrated that lamins and ribosomes function in the shared pathway to regulate cellular organization, we sought to determine the mechanistic relationship between these factors. Ribosomes are synthesized and assembled within the nucleolus before export to the cytoplasm (*14*). Previous studies in mammalian tissue culture cells showed that lamins play important roles in nucleolar function (*26*, *27*), but the effects of EDMD-associated lamin mutations on nucleolar organization and ribosome production in intact animal tissues remained unexplored. We hypothesized that lamin dysfunction disrupts cellular organization by interfering with ribosome biogenesis in the nucleolus.

**Fig. 4.**
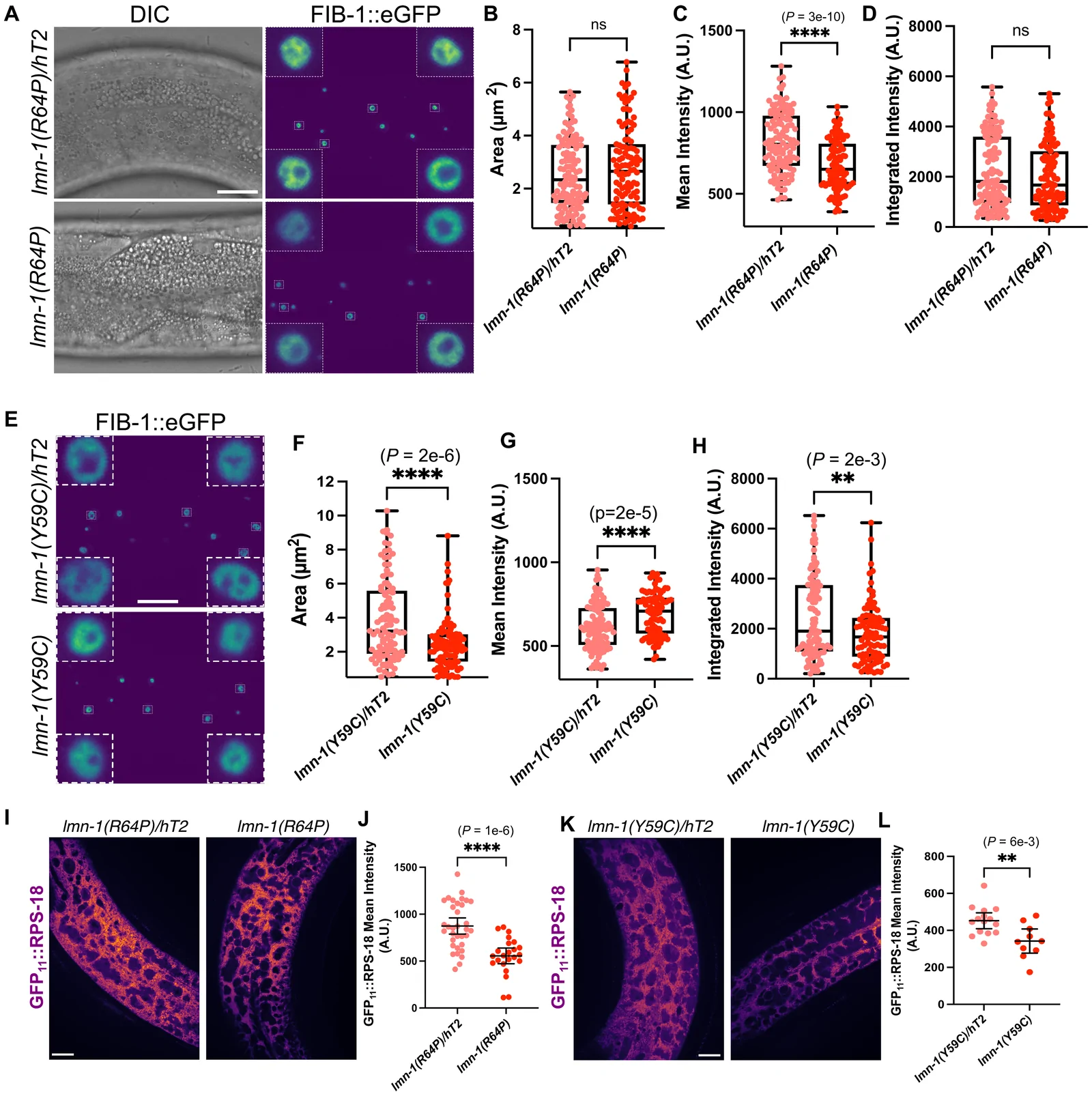
EDMD-associated *lmn-1* variants alter nucleolar organization and reduce ribosome abundance. (**A**) Representative spinning-disk confocal images of the hypodermis in day 1 adult *lmn-1(R64P)/hT2* and *lmn-1(R64P)* animals showing DIC (left) and FIB-1::eGFP fluorescence (right). Insets show magnified views of representative nucleoli. Fluorescence intensity is displayed using the viridis colormap. Scale bar, 10 μm. (**B** to **D**) Quantification of nucleolar area (B), mean FIB-1::eGFP fluorescence intensity (C), and integrated FIB-1::eGFP fluorescence intensity (D) in the indicated genotypes. Each dot represents one nucleolus (*n* = 126 and 111, left to right). (**E**) Representative spinning-disk confocal images of FIB-1::eGFP in the hypodermis of day 1 adult *lmn-1(Y59C)/hT2* and *lmn-1(Y59C)* animals. Insets show magnified views of representative nucleoli. Fluorescence intensity is displayed using the viridis colormap. Scale bar, 10 μm. (**F** to **H**) Quantification of nucleolar area (F), mean FIB-1::eGFP fluorescence intensity (G), and integrated FIB-1::eGFP fluorescence intensity (H) in the indicated genotypes. Each dot represents one nucleolus (*n* = 101 and 94 nucleoli from 10 worms per genotype, left to right). (**I** and **K**) Representative spinning-disk confocal images of hypodermal GFP_11_::RPS-18 fluorescence, reconstituted by split-GFP in day 1 adult *lmn-1(R64P)/*hT2 and *lmn-1(R64P)* animals (I), and *lmn-1(Y59C)/hT2* and *lmn-1(Y59C)* animals (K). Scale bar, 10 μm. (**J** and **L**) Mean GFP_11_::RPS-18 fluorescence intensity per animal in the indicated genotypes for R64P (J) and Y59C (L) backgrounds. Each dot represents one worm. Bars indicate mean ± 95% CI. Statistical significance was assessed by two-tailed Welch’s *t* test; exact p values are shown above each comparison. Asterisks denote significance as follows: ****P < 0.0001, ***P ≤ 0.001, **P ≤ 0.01, *P ≤ 0.05, and ns, *P* > 0.05.

To test this hypothesis, we first examined nucleolar organization in *lmn-1* mutant animals using FIB-1::eGFP, a fluorescently tagged fusion of the *C. elegans* fibrillarin ortholog, an essential nucleolar protein (*28*). Fibrillarin is a well-established marker of nucleolar activity whose levels correlate with ribosome biogenesis capacity across multiple species (*14*, *29*–*31*). Live imaging of nucleoli in the hypodermal syncytium allowed us to quantify nucleolar morphology and FIB-1::eGFP abundance (Fig. 4, A to H). In *lmn-1(R64P)* mutants, nucleolar size was comparable to *lmn-1(R64P)/hT2* controls (Fig. 4B), yet mean FIB-1::eGFP intensity was significantly reduced (Fig. 4C), without a corresponding change in total integrated fluorescence intensity (Fig. 4D). This pattern, reduced local concentration but preserved total signal, suggests that R64P alters the internal organization or compaction of nucleolar contents rather than overall FIB-1 abundance. In contrast, *lmn-1(Y59C)* mutants showed smaller nucleoli with elevated mean FIB-1::eGFP intensity but reduced total integrated fluorescence relative to *lmn-1(Y59C)/hT2* controls (Fig. 4, E to H), indicating an overall reduction in FIB-1 abundance concentrated into a smaller nucleolar volume. Thus, although both EDMD-associated lamin variants perturb nucleolar integrity, they do so in mechanistically distinct ways: R64P disrupts nucleolar organization without altering overall size or FIB-1 abundance, whereas Y59C produces smaller, denser nucleoli with reduced total FIB-1.

To further test whether lamin mutations perturb nucleolar organization, we analyzed a second independent nucleolar marker: NUCL-1::GFP, the endogenously tagged *C. elegans* ortholog of nucleolin (*32*). Consistent with the FIB-1::eGFP data, NUCL-1::GFP revealed nucleolar abnormalities in both *lmn-1(R64P)* and *lmn-1(Y59C)* mutant backgrounds (fig. S4), though the specific changes in nucleolar size and intensity differed between the two variants, consistent with the mechanistically distinct perturbations described above. Together, the concordant abnormalities detected with both FIB-1::eGFP and NUCL-1::GFP, markers with distinct nucleolar distributions, indicate that EDMD-associated lamin mutations broadly disrupt nucleolar organization rather than selectively affecting a single nucleolar component.

We next asked whether these nucleolar defects were accompanied by reduced ribosome abundance. Using endogenously tagged GFP11::RPS-18 visualized by split-GFP reconstitution in the hypodermis (*33*), we observed significant reductions in ribosomal fluorescence in both *lmn-1(R64P)* and *lmn-1(Y59C)* mutants relative to their respective balanced controls (Fig. 4, I to L). A second independent ribosomal marker, RPL-29::GFP (*33*), showed consistent reductions in hypodermal fluorescence intensity in both mutant backgrounds (fig. S5). Together, two independent nucleolar markers and two independent ribosomal markers point to the same conclusion: lamin dysfunction broadly perturbs nucleolar organization and reduces ribosome abundance.

### Activation of mTOR signaling partially restores cytoplasmic crowding in EDMD-associated lamin mutants

The results above suggested that impaired ribosome biogenesis may underlie the altered cytoplasmic biophysical properties of lamin mutants. If so, stimulating ribosome production should partially restore cytoplasmic crowding. We therefore tested whether activation of mTOR signaling could suppress the crowding defects of *lmn-1* mutants by depleting the components of the guanosine triphosphatase–activating protein activity toward Rags (GATOR) complex, specifically the GATOR1 components *nprl-2* or *nprl-3*, which negatively regulate mTORC1 activity (*34*), and quantifying hypodermal GEM diffusion.

**Fig. 5.**
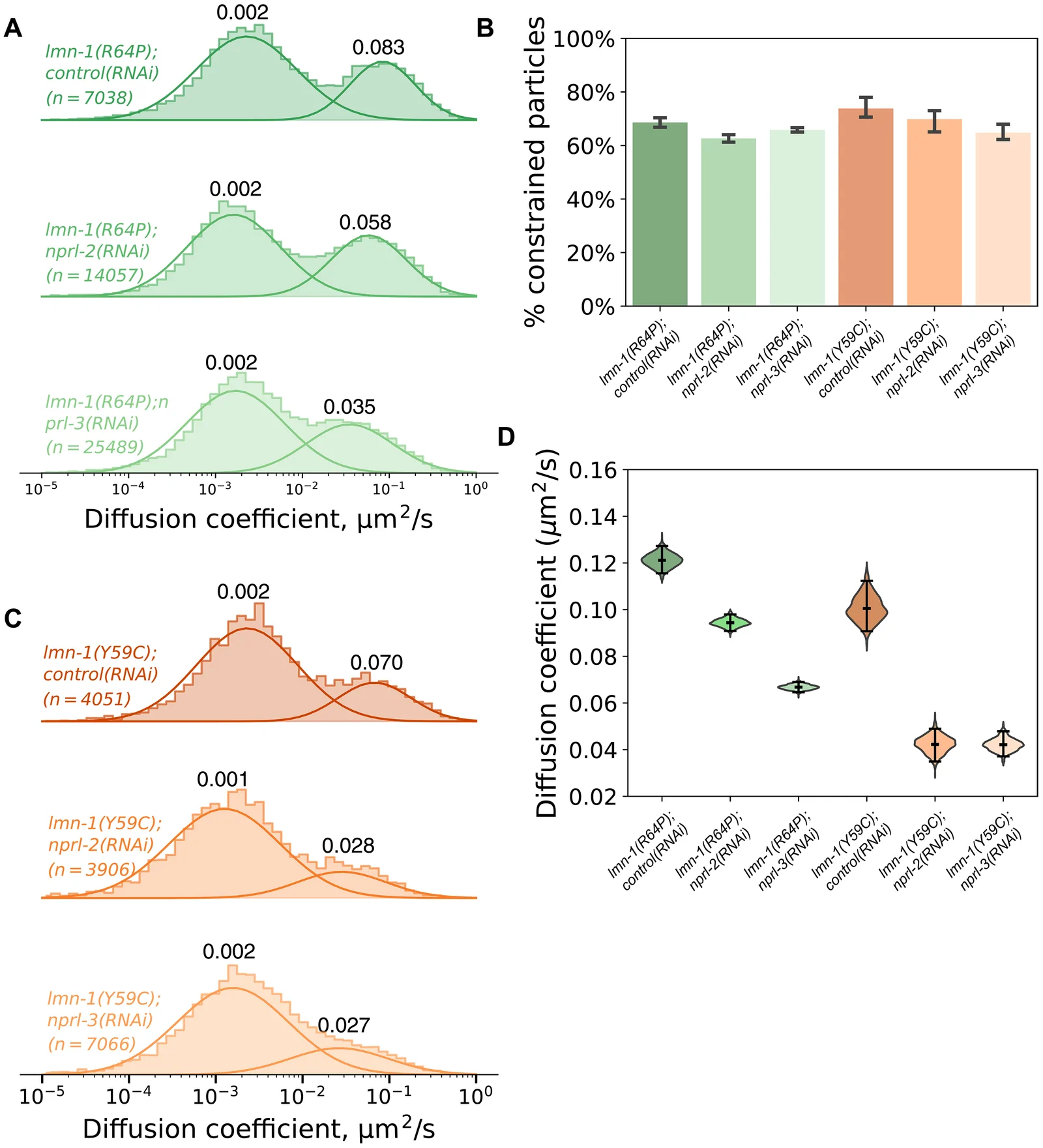
Activation of mTOR signaling by depletion of GATOR1 components partially restores cytoplasmic crowding in EDMD-associated *lmn-1* mutants. (**A** and **C**) GMM analysis of GEM *D*_eff_ distributions in the hypodermis of day 1 adult *lmn-1(R64P)* (A) and *lmn-1(Y59C)* (C) animals subjected to *control(RNAi)*, *nprl-2(RNAi)*, or *nprl-3(RNAi)*. Annotated values indicate the geometric mean of each identified peak. (**B** and **D**) Bootstrap analysis (1000 iterations) of GMM-derived GEM diffusion parameters in the hypodermis for the indicated conditions. Bar plots (B) show the percentage of the constrained GEM population; violin plots (D) show the mean *D*_eff_ values of the unconstrained GEM population. All values represent mean ± 95% CI.

In *lmn-1(R64P)* mutants, depletion of either *nprl-2* or *nprl-3* shifted the GEM diffusivity distributions toward lower values relative to *control(RNAi)* animals (Fig. 5A), consistent with partial restoration of cytoplasmic crowding. Bootstrap analysis showed this effect was driven primarily by a reduction in the effective diffusion coefficient of the unconstrained GEM population, with little change in the relative abundance of constrained fraction (Fig. 5, B and D). A similar pattern was observed in *lmn-1(Y59C)* mutants, where depletion of either *nprl-2* or *nprl-3* likewise reduced unconstrained GEM diffusivity relative to controls (Fig. 5, C and D). Together, these data suggest that mTOR activation partially restores mesoscale crowding in lamin mutants without substantially altering structural constraints.

Together, these findings support a model in which EDMD-associated lamin mutations impair a nucleolar-ribosomal axis that maintains cytoplasmic organization. By reducing nucleolar integrity and ribosome abundance, lamin dysfunction decreases cytoplasmic crowding and disrupts cellular organization, providing a molecular explanation for how nuclear lamina defects propagate to cause the broad cellular disorganization characteristic of EDMD.

### Cytoplasmic crowding defects are specific to EDMD-associated nuclear envelope proteins

**Fig. 6.**
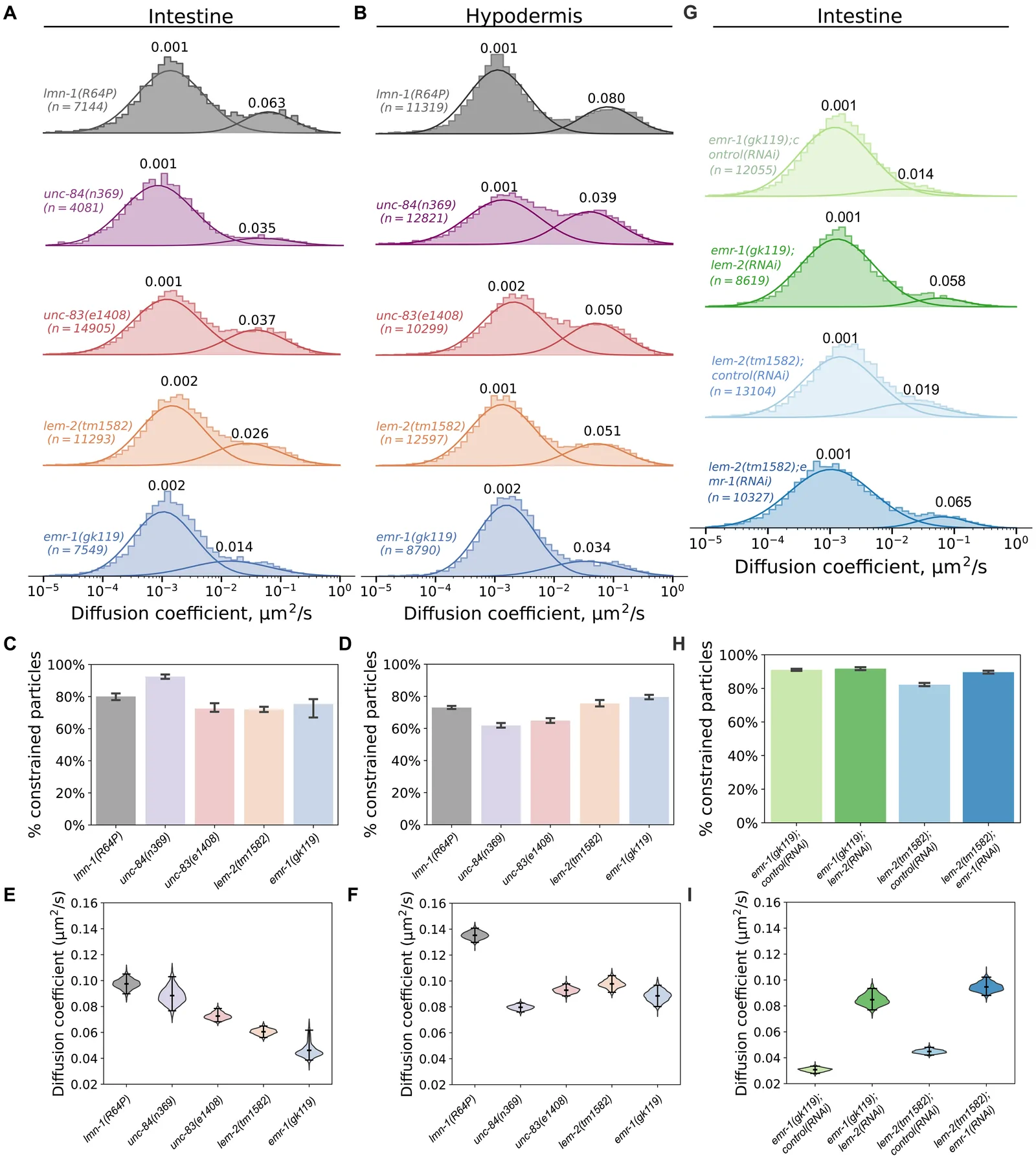
Combined loss of LEM-domain proteins mimics lamin dysfunction and disrupts cytoplasmic crowding. (**A**, **B**, and **G**) GMM analysis of GEM *D*_eff_ in the intestine (A and G) or hypodermis (B) of the indicated strains at day 1 of adulthood. Annotated values indicate the geometric mean of each identified peak. (**C** to **F**, **H**, and **I**) Bootstrap analysis (1000 iterations) of GMM-derived GEM diffusion parameters for the indicated strains. Bar plots (C, D, and H) show the percentage of the constrained GEM population in the intestine (C and H) or hypodermis (D); violin plots (E, F, and I) show the *D*_eff_ of the unconstrained GEM population in the intestine (E and I) or hypodermis (F). All values represent mean ± 95% CI.

To determine whether cytoplasmic organization defects were specific to EDMD-associated proteins or represent a general consequence of nuclear envelope disruption, we examined how various nuclear envelope protein mutations affect cytoplasmic biophysical properties using GEM particle tracking. In humans, loss of emerin alone causes X-linked EDMD (1). However, in *C. elegans*, the emerin ortholog EMR-1 functions redundantly with another LEM-domain protein, LEM-2, during early embryogenesis (*35*, *36*). Previous studies showed that codepletion of *emr-1* and *lem-2* recapitulate nuclear envelope defects and EDMD-like phenotypes observed in LMN-1–depleted animals (*19*, *35*, *37*). We found that single *lem-2(tm1582)* or *emr-1(gk119)* mutations produced minimal disruption to GEM diffusion patterns (Fig. 6, A to F, and fig. S3). However, *emr-1(gk119); lem-2(RNAi)* double mutants led to disruption of cytoplasmic crowding (Fig. 6, G to I). *emr-1(gk119); lem-2(RNAi)* animals had marked increased GEM diffusion coefficients, mirroring lamin mutants, demonstrating that combined loss of functionally redundant LEM-domain proteins resembles lamin dysfunction.

We also tested canonical LINC complex components, including the inner nuclear membrane Sad1/UNC-84 (SUN) domain protein UNC-84 and the outer nuclear membrane partner, the KASH protein UNC-83 (*38*–*40*). These proteins are core constituents of the LINC complex that mediates nuclear-cytoskeletal coupling and interact with lamin (*7*). In mammals, LINC complex components such as SUN1, SUN2, nesprin-1, and nesprin-2 have also been implicated in EDMD (*41*, *42*). However, animals carrying null mutations *unc-84(n369)* or *unc-83(e1408)* displayed only minimal alterations in cytoplasmic biophysical properties in both intestine and hypodermis (Fig. 6, A to F, and fig. S3).

These results indicate that disruption of individual nuclear envelope components does not substantially affect cytoplasmic organization. Across *unc-84(n369)*, *unc-83(e1408)*, *lem-2(tm1582)*, and *emr-1(gk119)* single mutants, the percentage of constrained particles remained largely unchanged (Fig. 6, C and D), and diffusion coefficients of unconstrained particles showed only modest variations (Fig. 6, E and F). Thus, cytoplasmic crowding defects are not a general consequence of nuclear envelope dysfunction but instead represent a specific hallmark of EDMD-associated protein disruption. The pronounced effect of combined emerin/LEM-domain perturbation, contrasted with the minimal impact of other nuclear envelope components loss, highlights the distinctive requirement for EDMD-linked proteins in maintaining cytoplasmic biophysical properties.

## DISCUSSION

**Fig. 7.**
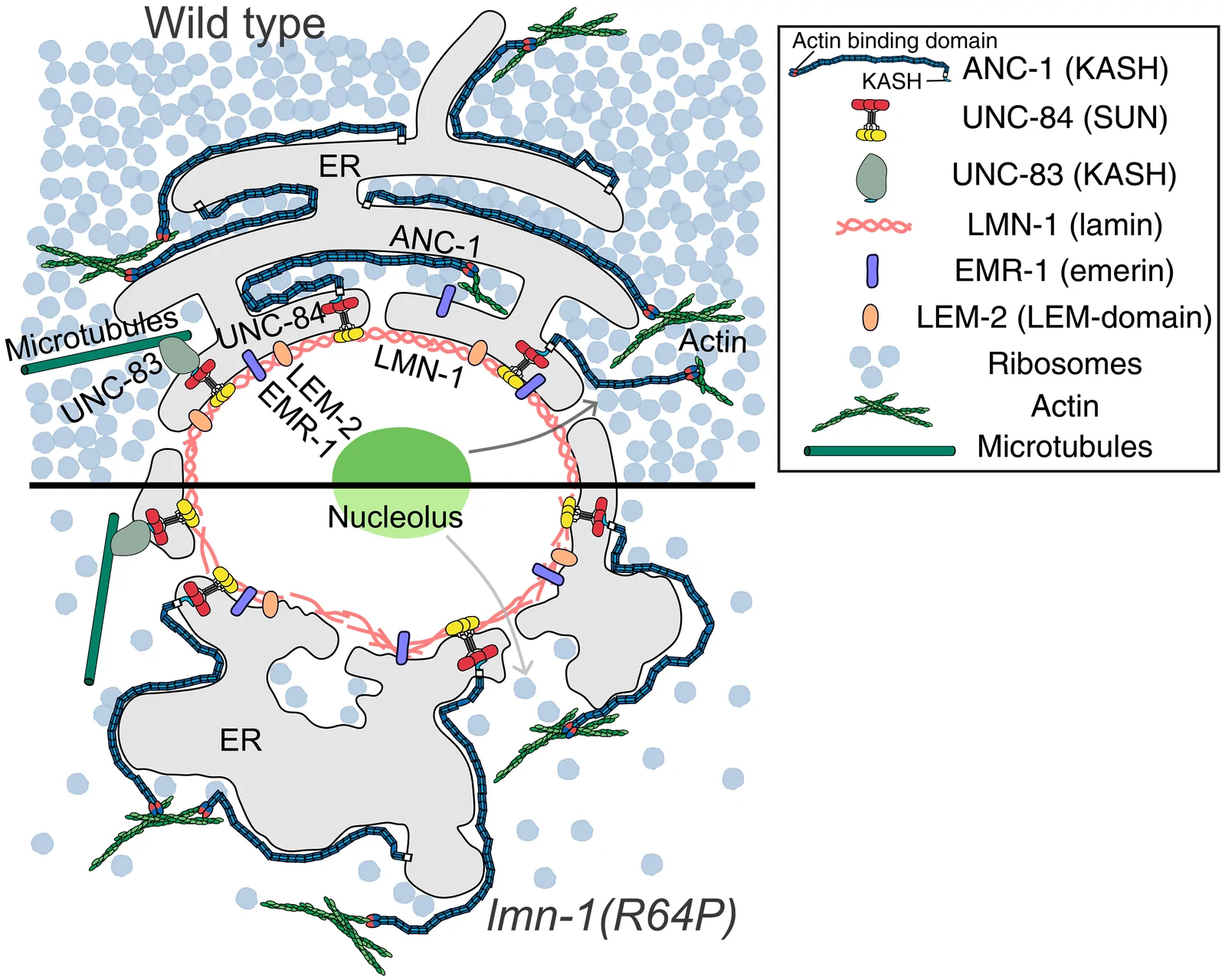
Nuclear lamina maintains cellular organization through a nucleolar-ribosome axis. Schematic depicting nuclear envelope architecture and the proposed pathway linking lamina integrity to cytoplasmic organization. The nuclear lamina (LMN-1/lamin), inner nuclear membrane proteins EMR-1 (emerin; LEM-domain) and LEM-2 (LEM-domain), and LINC complex components UNC-84 (SUN protein), ANC-1 (giant KASH protein), and UNC-83 (KASH protein) are shown. Ribosomes (blue circles), actin filaments (dark green), microtubules (teal), and the ER network (gray) are depicted as indicated in the legend. Top (wild-type): An intact nuclear lamina preserves nucleolar integrity, supporting ribosome biogenesis. High ribosome abundance sustains cytoplasmic macromolecular crowding, stabilizing ER network morphology and cellular organization. Bottom *[lmn-1(R64P)]*: Lamina dysfunction disrupts nucleolar organization, reduces ribosome abundance, and decreases cytoplasmic crowding, leading to ER network collapse and broad cellular disorganization.

This study uncovers a nuclear lamina-nucleolar-ribosomal axis through which defects in nuclear lamins propagate to the cytoplasm and disrupt cellular architecture. While previous studies identified connections between lamins and nucleolar function in cultured mammalian cells (*26*, *27*), our work shows in intact tissues that this relationship directly influences cytoplasmic biophysical properties and cellular organization. Using *C. elegans*, we show that EDMD-associated lamin variants reduce mesoscale cytoplasmic crowding, misposition nuclei, and collapse ER network complexity through effects on nucleolar organization and ribosome abundance (Fig. 7).

### Integration with existing laminopathy models

Two prevailing models have historically shaped explanations for how lamin mutations drive pathology: (i) lamina defects weaken nuclear architecture and mechanotransduction, rendering muscle nuclei vulnerable to mechanical stress and aberrant signaling; and (ii) altered chromosome organization disrupts gene regulation, impairing tissue-specific gene activation (*10*). Our nuclear lamina-nucleolar-ribosome framework complements these models by revealing how nuclear defects translate into widespread cytoplasmic disorganization, a prominent but less well characterized feature of EDMD.

Rather than replacing existing models, our results suggest that the nucleolar-ribosomal axis may act downstream of both mechanical and transcriptional defects; nucleolar dysfunction could arise from impaired nuclear mechanics, altered chromatin organization, or disrupted rRNA transcription (*27*, *43*–*50*). In support of this idea, we show that EDMD-linked *lmn-1* mutations disrupt nucleolar integrity and reduce ribosome abundance, thereby affecting cytoplasmic biophysical properties and linking nuclear lamina defects mechanistically to cytoplasmic disorganization. Our model accounts for cytoplasmic phenotypes that earlier frameworks have not fully addressed, including altered macromolecular crowding, disrupted ER architecture and organelle mispositioning. More broadly, these findings are consistent with emerging evidence that the cytoplasm exhibits widespread mesoscale organization and regulated material properties (*11*, *51*), and that nuclear envelope defects can perturb this organization through mechanisms extending beyond mechanics or transcription alone. Although our mTOR activation data support an upstream contribution of nucleolar-ribosomal dysfunction to the cytoplasmic phenotype, we cannot exclude the possibility that altered cytoplasmic organization feeds back on nucleolar state.

### Evolutionary conservation and clinical relevance

The nucleolar-ribosome axis is highly conserved across species (*52*, *53*), suggesting our findings in *C. elegans* are likely relevant to human disease. Mammalian lamins directly interact with nucleolar components and lamin mutations in patient-derived cells show nucleolar abnormalities (*26*, *27*), resembling our observations. The conservation of ribosome-dependent cytoplasmic crowding from *Escherichia coli* to mammals (*11*, *12*, *54*) further supports the translational relevance of our findings.

This conservation has important clinical implications. If nucleolar dysfunction and ribosome depletion were central to EDMD pathogenesis, therapeutic strategies could target multiple points in this pathway: enhancing ribosome biogenesis, modulating mTOR signaling to restore ribosome production, or using crowding agents to compensate for reduced ribosome levels. Such approaches could potentially benefit patients with mutations in either *LMNA* or *EMD*, providing broader therapeutic options than mutation-specific strategies.

### Tissue specificity and disease progression

The differential sensitivity between the intestine and hypodermis demonstrates the tissue-selective pathology characteristic of EDMD (*19*). Our finding that the *lmn-1(Y59C)* mutation affects GEM diffusion in the hypodermis but not the intestine illustrates how a ubiquitously expressed protein can produce tissue-restricted phenotypes. We propose that the hypodermal vulnerability reflects a combination of mechanical, biophysical, and epigenetic factors. First, the hypodermis occupies a structurally distinct mechanical environment: As a thin syncytium interposed between body wall muscle and the cuticle, it serves as the mechanical intermediary through which muscle contraction forces are transmitted to drive locomotion (*55*, *56*). This places hypodermal nuclei under repetitive mechanical strain during movement, in contrast to the relatively protected intestinal cells. Consistent with this, the EDMD mutation L535P disrupts nuclear mechanical responses specifically in muscle and associated tissues, but not in other cell types (*57*). Second, tissues differ in their biophysical resilience and ribosome abundance (*58*, *59*). The intestine maintains a ribosomal pool that appears to exceed its immediate translational demand. Even when ribosome biogenesis is partially compromised, the intestine may retain sufficient translational capacity to sustain normal cytoplasmic organization, whereas the hypodermis, which likely operates closer to its translational threshold, would be more vulnerable to nucleolar perturbation. Last, *lmn-1(Y59C)* specifically blocks the spatial relocation and activation of muscle gene promoters while leaving intestinal promoters unaffected (*19*), suggesting that epigenetic vulnerability is itself tissue-encoded (*60*). Together, tissues with high mechanical demands, limited translational reserve, and lamin-sensitive gene expression programs are predicted to be the earliest and most severely affected in EDMD (*61*), a prediction consistent with the clinical pattern of muscle and cardiac involvement in human disease.

### Mechanistic insights from genetic interactions

The phenotypic parallels between EDMD-associated lamin mutants and ribosome depletion are notable: Both disrupt nuclear positioning alter ER morphology and reduce cytoplasmic crowding. The greater severity of *lmn-1(R64P)* relative to *lmn-1(Y59C)* is consistent with the larger polymerization defects caused by the R64P substitution (*15*, *19*). Epistasis analyses further shows that lamin dysfunction and ribosome depletion act in a common pathway controlling cytoplasmic organization, whereas the giant KASH protein ANC-1 operates in a distinct pathway that organizes the cytoplasm by scaffolding the ER and imposing structural constraints (*12*). Together with the concordant reductions in nucleolar and ribosomal markers, these genetic relationships support a model in which EDMD-associated lamin mutations act upstream of ribosome biogenesis, coupling nuclear lamina dysfunction to cell-wide cytoplasmic disorganization.

### Resolving the mTOR paradox

A paradox emerges when considering the hyperactivation of mTORC1 signaling in laminopathies (*62*). Since mTORC1 is a central driver of ribosome biogenesis (*63*), it is unclear how lamin dysfunction produces both elevated mTOR signaling and reduced ribosome abundance. We suggest this additional contradiction is resolved by considering that lamins contribute directly to nucleolar structural integrity (*27*, *44*, *64*), and that pathogenic lamin variants trigger ER stress responses that suppress rRNA transcription (*65*, *66*), mechanisms that can limit ribosome production regardless of upstream mTOR activity. In this view, elevated mTOR signaling in laminopathies may represent a compensatory response to ribosomal insufficiency that is nonetheless unable to overcome nucleolar dysfunction. Consistent with this interpretation, our finding that further activating mTOR signaling through GATOR1 depletion partially restores cytoplasmic crowding indicates that the pathway retains functional capacity and that its output is limited at the nucleolar level rather than being fundamentally broken. This framework suggests that therapeutic strategies targeting nucleolar function or stress-induced suppression of rRNA transcription may be more effective complements to mTOR modulation than either approach alone.

### Technical advances and broader applications

The development of GEM-based nanorheology for intact tissues represents a substantial methodological advance with applications beyond laminopathy research. Similar nuclear GEM approaches were recently applied in mammalian cells to probe nuclear mechanical properties (*67*). By applying tissue-specific GEM expression, we learned that *C. elegans* maintains distinct cytoplasmic biophysical properties: Both crowded and constrained. Our study showed that EDMD-associated lamin mutations selectively reduce the crowding of unconstrained cytoplasmic particles, suggesting a role in regulating the concentration or organization of crowding agents rather than structural barriers. Given that ribosome concentration is a primary determinant of cytoplasmic crowding in multiple species (*12*, *54*, *68*), these results strongly suggest that lamin mutations impair ribosome levels, distribution, or assembly. This approach could be applied to study cytoplasmic biophysics in other diseases involving protein aggregation, metabolic disorders affecting ribosome function, or aging-related changes in cellular organization. Direct measurement of pre-rRNA synthesis rates, for example, by single-molecule fluorescence in situ hybridization, would provide further mechanistic resolution of the nucleolar-ribosomal axis and remains an important direction for future work. The ability to quantify cytoplasmic crowding in living animals provides a new biomarker for disease severity and therapeutic response that was previously unavailable.

### Therapeutic implications and future directions

Our finding that *emr-1* and *lem-2* function redundantly to regulate cytoplasmic crowding is relevant because these encode LEM domain proteins, including the *C. elegans* emerin ortholog (*36*), and mutations in human emerin cause X-linked EDMD (*1*). The *emr-1; lem-2* double mutants phenocopy lamin mutations in *C. elegans* embryonic mitosis and viability, further supporting that they function in a common pathway (*36*). Thus, both major EDMD-associated proteins, lamin and emerin, contribute to regulating cytoplasmic biophysical properties.

The partial functional redundancy between *emr-1* and *lem-2* may explain why EDMD pathology is variable and tissue-specific, as functional compensation between nuclear envelope proteins could mask defects in most tissues. The identification of the lamina-nucleolar-ribosome axis as a common pathway affected in both lamin and emerin associated forms of EDMD suggests potential therapeutic opportunities. GEM-based nanorheology could serve as a useful tool for monitoring cellular organization in preclinical studies.

A limitation of this study is that all experiments were performed in *C. elegans*, which, while disease relevant, represents a simplified metazoan context. However, this system uniquely enables quantitative in vivo analysis of cellular biophysics with single-cell resolution, an approach not yet feasible in mammalian models. Future work will be needed to determine how the lamina-nucleolar-ribosomal axis is implemented in mammalian tissues and to identify additional molecular factors that modulate cytoplasmic organization. Such efforts will further clarify how nuclear architecture governs cellular organization across species and disease contexts.

In conclusion, this work reveals a mechanistic pathway through which nuclear lamina defects impair nucleolar function and ribosome biogenesis, propagating to disrupt cytoplasmic organization and whole-cell biophysics. The use of in vivo quantitative GEM-based nanorheology provides a powerful approach for measuring cellular material properties in intact tissues. Together, our findings show that both major EDMD-associated proteins, lamin and emerin, preserve cellular organization by sustaining cytoplasmic crowding, offering molecular insight into disease mechanisms and suggesting therapeutic strategies aimed at restoring ribosome function.

## MATERIALS AND METHODS

### *C. elegans* strains and maintenance

All *C. elegans* strains used in this study are listed in table S1. Worms were maintained on standard nematode growth medium (NGM) agar plates seeded with *E. coli* strain OP50 at 23°C (*69*). For all experiments, synchronized animals were cultured to young adult stage (24 hours post-L4) under nonstarved conditions. Experimental animals were maintained on fresh plates for a minimum of two generations before experimentation to ensure consistent physiological conditions.

### RNA interference

Gene knockdown was performed using standard feeding RNAi methodology (*70*, *71*). Bacterial strains harboring specific RNAi clones were obtained from the Ahringer RNAi library (Source Bioscience, Nottingham, UK) and verified by Sanger sequencing before use. Individual bacterial colonies were picked from fresh plates and grown overnight at 37°C in 1 ml of LB medium supplemented with carbenicillin (100 μg/ml) in 15-ml snap-cap tubes on a rotary shaker. Cultures were diluted 1:40 in 2 ml of fresh LB-carbenicillin medium and grown for 3 hours at 37°C. For dsRNA induction, 2 ml of prewarmed LB medium containing carbenicillin (100 μg/ml) and 1 mM isopropyl β-d-1-thiogalactopyranoside (IPTG) was added to achieve a final IPTG concentration of 0.5 mM, followed by incubation for 3 to 4 hours at 37°C. Induced bacterial cultures were spotted onto NGM agar plates containing carbenicillin (25 μg/ml) and 1 mM IPTG. Synchronized L2-L3 stage larvae were transferred to RNAi feeding plates and maintained at 23°C for 48 hours until reaching young adult stage. To achieve optimal GEM expression levels for single-particle tracking in hypodermal tissue, L3 stage worms expressing GEM-EGFP were fed bacteria expressing dsRNA targeting *gfp* for 48 hours until egg-laying commenced (*12*). L1 progenies were then transferred to standard NGM plates with OP50 bacteria and cultured until imaging age. This protocol reduced GEM concentration to appropriate levels for tracking analysis.

### GEM detection and motion analysis

GEMs were imaged as previously reported (*12*). In brief, live imaging was performed using a Nikon Ti2 inverted microscope (Nikon Instruments, Melville, NY) equipped with a Yokogawa CSU-X1 spinning disk confocal scanner unit (Yokogawa Electric Corporation, Tokyo, Japan) and a Hamamatsu ORCA-Flash4.0 LT3 Digital sCMOS camera (Hamamatsu Photonics, Shizuoka, Japan). Images were acquired using a Nikon Plan Apo λ 100× oil immersion objective [numerical aperture (NA), 1.45] at 1060 × 1568 pixels resolution (0.065 μm/pixel). GEMs were visualized using 488-nm laser excitation at 100% power with 20 ms exposure time per frame in continuous acquisition mode (no interframe interval). Time series data were collected at 50 Hz acquisition rate for motion analysis. GEM particle detection and tracking were performed using published methods (*12*). In brief, trajectories shorter than 10 frames (0.2 s) were excluded from MSD analysis to ensure statistical reliability. For each valid trajectory, the effective diffusion coefficient (*D*_eff_) was calculated by linear fitting of the first five data points (100 ms total duration) of the MSD curve. Two-component Gaussian mixture models were fitted to *D*_eff_ distributions to resolve subpopulations of constrained and unconstrained particles. Parameter distributions and confidence intervals were estimated using bootstrap resampling with 1000 iterations (*12*).

### ER imaging and morphological analysis

ER networks were visualized using the mKate2::TRAM-1 fluorescent marker (strain UD756; table S1). Images were acquired using the Nikon Ti2 microscope system described above with 561-nm laser excitation at 50% power, 100 ms exposure time, and identical objective and camera configurations. ER occupancy and complexity analyses were carried out as previously described (*12*, *25*). In brief, images underwent background subtraction and noise reduction before binary segmentation using Otsu’s thresholding method. Two morphometric parameters were quantified: (i) ER occupancy, defined as the ratio of ER-positive area to total cellular area, and (ii) ER network complexity, calculated as the perimeter-to-area ratio (*P*^2^/4π*A*), where *P* represents the total perimeter and *A* represents the total area of ER-positive regions.

### Nuclear positioning measurements

Hypodermal nuclei were visualized using nuclear-localized GFP expressed from the integrated transgene *ycIs10[p_col-10_::nls::*GFP*::lacZ]* (*22*). Young adult worms were cultured on either standard NGM plates with OP50 *E. coli* (controls) or RNAi feeding plates with HT115 *E. coli* expressing target dsRNA. Microscope slides were prepared as introduced previously (*22*). Nuclear positioning was assessed using an Andor BC43 microscope with 488-nm laser excitation and a 60×/1.42 oil immersion objective. To ensure systematic counting, the anus and pharynx of each worm were positioned in the same focal plane. Nuclear clustering was quantified by counting GFP-positive hypodermal nuclei in direct contact with neighboring nuclei (*22*, *23*). Contacts along the perpendicular axis were excluded from analysis as the nuclear marker does not distinguish seam cell nuclei from hyp7 nuclear clusters. The percentage of clustered nuclei was calculated as the number of nuclei in contact divided by the total number of nuclei counted per animal.

### Nucleolar measurements

Nucleoli in the hypodermal tissues were visualized using FIB-1::eGFP (*72*) or the endogenously tagged NUCL-1::GFP (*32*) in the indicated strains. Worms were synchronized to L4 stage and imaged 24 hours later as young adults. Animals were immobilized using 10 μM tetramisole in M9 buffer on 2% agarose pads using standard procedures (*22*). Confocal microscopy on BC43 was performed using an Andor BC43 microscope with 488-nm laser excitation (3.0% laser power, 100 ms exposure time) and a 60×/1.42 oil immersion objective. Bright-field imaging was performed concurrently for tissue localization. Nucleolar segmentation was performed using Otsu’s automatic thresholding method (*73*) in Fiji/ImageJ software, with size filters applied (0.5 μm^2^ to infinity) to exclude artifacts. Regions of interest encompassing all identified nucleolar objects were generated and applied to original raw images for fluorescence intensity measurements. Mean fluorescence intensity was measured for each segmented nucleolus.

### Ribosome visualization and analysis

Ribosomes were visualized using either GFP11::RPS-18, detected by split-GFP reconstitution using the endogenous allele *yc113[GFP_11_::rps-18]* IV (*12*), or an RPL-29::GFP single copy insertion in the indicated strains (*33*). The complementary GFP_1-10_ construct was expressed in hypodermal tissue using the transgene *juEx5375[*p*_*col-19*_::GFP*_*(1-10)*_*+ *p*_*ttx-3*_::RFP]* (*33*), enabling reconstitution of functional GFP fluorescence specifically in the hypodermis. Young adult worms were immobilized using 10 μM tetramisole in M9 buffer on 2% agarose pads as described above. Images were acquired using the above-described Nikon Ti2 microscope system with a 100× oil immersion objective (NA 1.45) at 1060 × 1568 pixel resolution (0.065 μm/pixel). For GFP_11_::RPS-18, fluorescence was captured using 488-nm laser excitation at 30% power with 50 ms exposure time. Because RPL-29::GFP is broadly expressed throughout the animal (*33*), the hypodermis was first identified by morphology before image acquisition. A single optical section through this tissue was then captured for fluorescence quantification (488-nm laser excitation, 5% laser power, 500 ms exposure). Hypodermal cell boundaries were manually delineated to define regions of interest for fluorescence intensity measurements. Average ribosome fluorescence intensity was quantified within the hypodermal syncytium.

### Statistical analyses

All statistical analyses were performed using Prism GraphPad software (GraphPad Software Inc.). Sample sizes (*n*), degree of freedom and exact *P* values are reported in the figure legends. Nuclear positioning defects were analyzed using Welch’s analysis of variance (ANOVA) to account for unequal variances, followed by Dunnett’s T3 post hoc test for multiple comparisons. ER morphological defects were analyzed using Kruskal-Wallis followed by Dunnett’s T3 post hoc test for multiple comparisons. For nucleolus and ribosome quantification, two-tailed Welch’s t-test was performed. All statistical significance is indicated by asterisks (****P < 0.0001, ***P ≤ 0.001,**P ≤ 0.01, *P ≤ 0.05, and ns: *P* > 0.05) with exact *P* values shown above each comparison in each legend.

## References

[1] S. A. Heller, R. Shih, R. Kalra, P. B. Kang, Emery-Dreifuss muscular dystrophy. Muscle Nerve 61, 436–448 (2020). 31840275 10.1002/mus.26782 PMC7154529

[2] J. M. Sobo, N. S. Alagna, S. X. Sun, K. L. Wilson, K. L. Reddy, Lamins: The backbone of the nucleocytoskeleton interface. Curr. Opin. Cell Biol. 86, 102313 (2024). 38262116 10.1016/j.ceb.2023.102313

[3] A. Brachner, R. Foisner, Evolvement of LEM proteins as chromatin tethers at the nuclear periphery. Biochem. Soc. Trans. 39, 1735–1741 (2011). 22103517 10.1042/BST20110724 PMC4333761

[4] L. J. Barton, A. A. Soshnev, P. K. Geyer, Networking in the nucleus: A spotlight on LEM-domain proteins. Curr. Opin. Cell Biol. 34, 1–8 (2015). 25863918 10.1016/j.ceb.2015.03.005 PMC4522374

[5] E. S. Folker, M. K. Baylies, Nuclear positioning in muscle development and disease. Front. Physiol. 4, 363 (2013). 24376424 10.3389/fphys.2013.00363 PMC3859928

[6] M. J. Puckelwartz, E. Kessler, Y. Zhang, D. Hodzic, K. N. Randles, G. Morris, J. U. Earley, M. Hadhazy, J. M. Holaska, S. K. Mewborn, P. Pytel, E. M. McNally, Disruption of nesprin-1 produces an Emery Dreifuss muscular dystrophy-like phenotype in mice. Hum. Mol. Genet. 18, 607–620 (2009). 19008300 10.1093/hmg/ddn386 PMC2722216

[7] C. R. Bone, E. C. Tapley, M. Gorjánácz, D. A. Starr, The *Caenorhabditis elegans* SUN protein UNC-84 interacts with lamin to transfer forces from the cytoplasm to the nucleoskeleton during nuclear migration. Mol. Biol. Cell 25, 2853–2865 (2014). 25057012 10.1091/mbc.E14-05-0971 PMC4161519

[8] F. Haque, D. J. Lloyd, D. T. Smallwood, C. L. Dent, C. M. Shanahan, A. M. Fry, R. C. Trembath, S. Shackleton, SUN1 interacts with nuclear lamin A and cytoplasmic nesprins to provide a physical connection between the nuclear lamina and the cytoskeleton. Mol. Cell. Biol. 26, 3738–3751 (2006). 16648470 10.1128/MCB.26.10.3738-3751.2006 PMC1488999

[9] M. A. Collins, T. R. Mandigo, J. M. Camuglia, G. A. Vazquez, A. J. Anderson, C. H. Hudson, J. L. Hanron, E. S. Folker, Emery-Dreifuss muscular dystrophy-linked genes and centronuclear myopathy-linked genes regulate myonuclear movement by distinct mechanisms. Mol. Biol. Cell 28, 2303–2317 (2017). 28637766 10.1091/mbc.E16-10-0721 PMC5555658

[10] X. Wong, C. L. Stewart, The laminopathies and the insights they provide into the structural and functional organization of the nucleus. Annu. Rev. Genomics Hum. Genet. 21, 263–288 (2020). 32428417 10.1146/annurev-genom-121219-083616

[11] M. Delarue, G. P. Brittingham, S. Pfeffer, I. V. Surovtsev, S. Pinglay, K. J. Kennedy, M. Schaffer, J. I. Gutierrez, D. Sang, G. Poterewicz, J. K. Chung, J. M. Plitzko, J. T. Groves, C. Jacobs-Wagner, B. D. Engel, L. J. Holt, mTORC1 controls phase separation and the biophysical properties of the cytoplasm by tuning crowding. Cell 174, 338–349.e20 (2018). 29937223 10.1016/j.cell.2018.05.042 PMC10080728

[12] X. Ding, H. Hao, D. Elnatan, P. N. Alinaya, S. Kalra, A. Kaur, S. Kumari, L. J. Holt, G. W. G. Luxton, D. A. Starr, Giant KASH proteins and ribosomes establish distinct cytoplasmic biophysical properties in vivo. Sci. Adv. 11, eadx0952 (2025). 40929259 10.1126/sciadv.adx0952 PMC12422203

[13] C. Alfano, Y. Fichou, K. Huber, M. Weiss, E. Spruijt, S. Ebbinghaus, G. De Luca, M. A. Morando, V. Vetri, P. A. Temussi, A. Pastore, Molecular crowding: The history and development of a scientific paradigm. Chem. Rev. 124, 3186–3219 (2024). 38466779 10.1021/acs.chemrev.3c00615 PMC10979406

[14] D. L. J. Lafontaine, J. A. Riback, R. Bascetin, C. P. Brangwynne, The nucleolus as a multiphase liquid condensate. Nat. Rev. Mol. Cell Biol. 22, 165–182 (2021). 32873929 10.1038/s41580-020-0272-6

[15] N. Wiesel, A. Mattout, S. Melcer, N. Melamed-Book, H. Herrmann, O. Medalia, U. Aebi, Y. Gruenbaum, Laminopathic mutations interfere with the assembly, localization, and dynamics of nuclear lamins. Proc. Natl. Acad. Sci. U.S.A. 105, 180–185 (2008). 18162544 10.1073/pnas.0708974105 PMC2224182

[16] J. Liu, T. Rolef Ben-Shahar, D. Riemer, M. Treinin, P. Spann, K. Weber, A. Fire, Y. Gruenbaum, Essential roles for Caenorhabditis elegans lamin gene in nuclear organization, cell cycle progression, and spatial organization of nuclear pore complexes. Mol. Biol. Cell 11, 3937–3947 (2000). 11071918 10.1091/mbc.11.11.3937 PMC15048

[17] E. F. Gregory, S. Kalra, T. Brock, G. Bonne, G. W. G. Luxton, C. Hopkins, D. A. Starr, Caenorhabditis elegans models for striated muscle disorders caused by missense variants of human LMNA. PLOS Genet. 19, e1010895 (2023). 37624850 10.1371/journal.pgen.1010895 PMC10484454

[18] M. L. Edgely, D. L. Baillie, D. L. Riddle, A. M. Rose, Genetic balancers. WormBook 10.1895/wormbook.1.89.1 (2006). 10.1895/wormbook.1.89.1 PMC4781404 18050450

[19] A. Mattout, B. L. Pike, B. D. Towbin, E. M. Bank, A. Gonzalez-Sandoval, M. B. Stadler, P. Meister, Y. Gruenbaum, S. M. Gasser, An EDMD mutation in C. elegans lamin blocks muscle-specific gene relocation and compromises muscle integrity. Curr. Biol. 21, 1603–1614 (2011). 21962710 10.1016/j.cub.2011.08.030

[20] H. Hao, S. Kalra, L. E. Jameson, L. A. Guerrero, N. E. Cain, J. Bolivar, D. A. Starr, The Nesprin-1/−2 ortholog ANC-1 regulates organelle positioning in C. elegans independently from its KASH or actin-binding domains. eLife 10, e61069 (2021). 33860766 10.7554/eLife.61069 PMC8139857

[21] D. A. Starr, M. Han, Role of ANC-1 in tethering nuclei to the actin cytoskeleton. Science 298, 406–409 (2002). 12169658 10.1126/science.1075119

[22] H. N. Fridolfsson, L. A. Herrera, J. N. Brandt, N. E. Cain, G. J. Hermann, D. A. Starr, Genetic analysis of nuclear migration and anchorage to study linc complexes during development of Caenorhabditis elegans. Methods Mol. Biol. 1840, 163–180 (2018). 30141045 10.1007/978-1-4939-8691-0_13 PMC6410724

[23] N. E. Cain, Z. Jahed, A. Schoenhofen, V. A. Valdez, B. Elkin, H. Hao, N. J. Harris, L. A. Herrera, B. M. Woolums, M. R. K. Mofrad, G. W. G. Luxton, D. A. Starr, Conserved SUN-KASH interfaces mediate LINC complex-dependent nuclear movement and positioning. Curr. Biol. 28, 3086–3097.e4 (2018). 30245107 10.1016/j.cub.2018.08.001 PMC6219386

[24] H. T. Perkins, V. Allan, Intertwined and finely balanced: Endoplasmic reticulum morphology, dynamics, function, and diseases. Cells 10, 2341 (2021). 34571990 10.3390/cells10092341 PMC8472773

[25] A. Watson, Perimetric complexity of binary digital images. Math. J. 14, 10.3888/tmj.14-5 (2012).

[26] A. Buchwalter, M. W. Hetzer, Nucleolar expansion and elevated protein translation in premature aging. Nat. Commun. 8, 328 (2017). 28855503 10.1038/s41467-017-00322-z PMC5577202

[27] C. Martin, S. Chen, A. Maya-Mendoza, J. Lovric, P. F. G. Sims, D. A. Jackson, Lamin B1 maintains the functional plasticity of nucleoli. J. Cell Sci. 122, 1551–1562 (2009). 19383719 10.1242/jcs.046284 PMC2722682

[28] L.-W. Lee, C.-C. Lee, C.-R. Huang, S. J. Lo, The nucleolus of Caenorhabditis elegans. J. Biomed. Biotechnol. 2012, 601274 (2012). 22577294 10.1155/2012/601274 PMC3345250

[29] K. Newton, E. Petfalski, D. Tollervey, J. F. Cáceres, Fibrillarin is essential for early development and required for accumulation of an intron-encoded small nucleolar RNA in the mouse. Mol. Cell. Biol. 23, 8519–8527 (2003). 14612397 10.1128/MCB.23.23.8519-8527.2003 PMC262675

[30] C. P. Brangwynne, T. J. Mitchison, A. A. Hyman, Active liquid-like behavior of nucleoli determines their size and shape in *Xenopus laevis* oocytes. Proc. Natl. Acad. Sci. U.S.A. 108, 4334–4339 (2011). 21368180 10.1073/pnas.1017150108 PMC3060270

[31] D. Korčeková, A. Gombitová, I. Raška, D. Cmarko, C. Lanctôt, Nucleologenesis in the Caenorhabditis elegans embryo. PLOS ONE 7, e40290 (2012). 22768349 10.1371/journal.pone.0040290 PMC3388055

[32] E. L. Spaulding, D. L. Updike, C. elegans nucleolar RG repeats are sufficient for nucleolar accumulation but insufficient for sub-nucleolar compartmentalization. bioRxiv 2024.12.19.629445 [Preprint] (2024). 10.1101/2024.12.19.629445.

[33] K. Noma, A. Goncharov, M. H. Ellisman, Y. Jin, Microtubule-dependent ribosome localization in C. elegans neurons. eLife 6, e26376 (2017). 28767038 10.7554/eLife.26376 PMC5577916

[34] L. Bar-Peled, L. Chantranupong, A. D. Cherniack, W. W. Chen, K. A. Ottina, B. C. Grabiner, E. D. Spear, S. L. Carter, M. Meyerson, D. M. Sabatini, A tumor suppressor complex with GAP Activity for the Rag GTPases that signal amino acid sufficiency to mTORC1. Science 340, 1100–1106 (2013). 23723238 10.1126/science.1232044 PMC3728654

[35] J. Liu, K. K. Lee, M. Segura-Totten, E. Neufeld, K. L. Wilson, Y. Gruenbaum, MAN1 and emerin have overlapping function(s) essential for chromosome segregation and cell division in *Caenorhabditis elegans*. Proc. Natl. Acad. Sci. U.S.A. 100, 4598–4603 (2003). 12684533 10.1073/pnas.0730821100 PMC153601

[36] R. Barkan, A. J. Zahand, K. Sharabi, A. T. Lamm, N. Feinstein, E. Haithcock, K. L. Wilson, J. Liu, Y. Gruenbaum, Ce-emerin and LEM-2: Essential roles in *Caenorhabditis elegans* development, muscle function, and mitosis. Mol. Biol. Cell 23, 543–552 (2012). 22171324 10.1091/mbc.E11-06-0505 PMC3279384

[37] O. Cohen-Fix, P. Askjaer, Cell biology of the *Caenorhabditis elegans* nucleus. Genetics 205, 25–59 (2017). 28049702 10.1534/genetics.116.197160 PMC5216270

[38] C. J. Malone, W. D. Fixsen, H. R. Horvitz, M. Han, UNC-84 localizes to the nuclear envelope and is required for nuclear migration and anchoring during *C. elegans* development. Development 126, 3171–3181 (1999). 10375507 10.1242/dev.126.14.3171

[39] M. D. McGee, R. Rillo, A. S. Anderson, D. A. Starr, UNC-83 is a KASH protein required for nuclear migration and is recruited to the outer nuclear membrane by a physical interaction with the SUN protein UNC-84. Mol. Biol. Cell 17, 1790–1801 (2006). 16481402 10.1091/mbc.E05-09-0894 PMC1415293

[40] D. A. Starr, A network of nuclear envelope proteins and cytoskeletal force generators mediates movements of and within nuclei throughout *Caenorhabditis elegans* development. Exp. Biol. Med. 244, 1323–1332 (2019). 10.1177/1535370219871965 PMC6880151 31495194

[41] Q. Zhang, C. Bethmann, N. F. Worth, J. D. Davies, C. Wasner, A. Feuer, C. D. Ragnauth, Q. Yi, J. A. Mellad, D. T. Warren, M. A. Wheeler, J. A. Ellis, J. N. Skepper, M. Vorgerd, B. Schlotter-Weigel, P. L. Weissberg, R. G. Roberts, M. Wehnert, C. M. Shanahan, Nesprin-1 and -2 are involved in the pathogenesis of Emery Dreifuss muscular dystrophy and are critical for nuclear envelope integrity. Hum. Mol. Genet. 16, 2816–2833 (2007). 17761684 10.1093/hmg/ddm238

[42] P. Meinke, E. Mattioli, F. Haque, S. Antoku, M. Columbaro, K. R. Straatman, H. J. Worman, G. G. Gundersen, G. Lattanzi, M. Wehnert, S. Shackleton, Muscular dystrophy-associated SUN1 and SUN2 variants disrupt nuclear-cytoskeletal connections and myonuclear organization. PLOS Genet. 10, e1004605 (2014). 25210889 10.1371/journal.pgen.1004605 PMC4161305

[43] O. Moujaber, N. Omran, M. Kodiha, B. Pié, E. Cooper, J. F. Presley, U. Stochaj, Data on the association of the nuclear envelope protein Sun1 with nucleoli. Data Brief 13, 115–123 (2017). 28580408 10.1016/j.dib.2017.05.028 PMC5447391

[44] A. S. Gupta, K. Sengupta, Lamin B2 modulates nucleolar morphology, dynamics, and function. Mol. Cell. Biol. 37, e00274–17 (2017). 10.1128/MCB.00274-17 PMC5705821 28993479

[45] J. Berry, S. C. Weber, N. Vaidya, M. Haataja, C. P. Brangwynne, RNA transcription modulates phase transition-driven nuclear body assembly. Proc. Natl. Acad. Sci. U.S.A. 112, E5237–E5245 (2015). 26351690 10.1073/pnas.1509317112 PMC4586886

[46] N. Kodan, R. Hussaini, S. C. Weber, J. Kondev, L. Mohapatra, Transcription-templated assembly of the nucleolus in the *Caenorhabditis elegans* embryo. Proc. Natl. Acad. Sci. U.S.A. 122, e2411964122 (2025). 40445759 10.1073/pnas.2411964122 PMC12146744

[47] H. Falahati, B. Pelham-Webb, S. Blythe, E. Wieschaus, Nucleation by rRNA dictates the precision of nucleolus assembly. Curr. Biol. 26, 277–285 (2016). 26776729 10.1016/j.cub.2015.11.065 PMC5866055

[48] J. Z. Zhao, J. Xia, C. P. Brangwynne, Chromatin compaction during confined cell migration induces and reshapes nuclear condensates. Nat. Commun. 15, 9964 (2024). 39557835 10.1038/s41467-024-54120-5 PMC11574006

[49] J. Xia, J. Z. Zhao, A. R. Strom, C. P. Brangwynne, Chromatin heterogeneity modulates nuclear condensate dynamics and phase behavior. Nat. Commun. 16, 6406 (2025). 40645941 10.1038/s41467-025-60771-9 PMC12254321

[50] S. A. Quinodoz, L. Jiang, A. A. Abu-Alfa, T. J. Comi, H. Zhao, Q. Yu, L. W. Wiesner, J. F. Botello, A. Donlic, E. Soehalim, P. Bhat, C. Zorbas, L. Wacheul, A. Košmrlj, D. L. J. Lafontaine, S. Klinge, C. P. Brangwynne, Mapping and engineering RNA-driven architecture of the multiphase nucleolus. Nature 644, 557–566 (2025). 40604277 10.1038/s41586-025-09207-4 PMC12350172

[51] F. C. Keber, T. Nguyen, A. Mariossi, C. P. Brangwynne, M. Wühr, Evidence for widespread cytoplasmic structuring into mesoscale condensates. Nat. Cell Biol. 26, 346–352 (2024). 38424273 10.1038/s41556-024-01363-5 PMC10981939

[52] C. C. Correll, J. Bartek, M. Dundr, The nucleolus: A multiphase condensate balancing ribosome synthesis and translational capacity in health, aging and ribosomopathies. Cells 8, 869 (2019). 31405125 10.3390/cells8080869 PMC6721831

[53] D. Ponti, The nucleolus: A central hub for ribosome biogenesis and cellular regulatory signals. Int. J. Mol. Sci. 26, 4174 (2025). 40362410 10.3390/ijms26094174 PMC12071546

[54] V. R. Gade, S. Heinrich, M. Paloni, P. A. Gómez-García, A. Dzanko, A. Oswald, D. Marchand, S. Khawaja, A. Barducci, K. Weis, Polysomes and mRNA control the biophysical properties of the eukaryotic cytoplasm. Cell Rep. 44, 116204 (2025). 40892542 10.1016/j.celrep.2025.116204

[55] K. Gieseler, Development, structure, and maintenance of C. elegans body wall muscle. WormBook 10.1895/wormbook.1.81.2 (2017). 10.1895/wormbook.1.81.2 PMC5410635 27555356

[56] B. C. Petzold, S.-J. Park, P. Ponce, C. Roozeboom, C. Powell, M. B. Goodman, B. L. Pruitt, Caenorhabditis elegans body mechanics are regulated by body wall muscle tone. Biophys. J. 100, 1977–1985 (2011). 21504734 10.1016/j.bpj.2011.02.035 PMC3077690

[57] N. Zuela, M. Zwerger, T. Levin, O. Medalia, Y. Gruenbaum, Impaired mechanical response of an EDMD mutation leads to motility phenotypes that are repaired by loss of prenylation. J. Cell Sci. 129, 1781–1791 (2016). 27034135 10.1242/jcs.184309

[58] H. M. Somers, J. H. Fuqua, F. X. A. Bonnet, J. A. Rollins, Quantification of tissue-specific protein translation in whole C. elegans using O-propargyl-puromycin labeling and fluorescence microscopy. Cell Rep. Methods 2, 100203 (2022). 35497499 10.1016/j.crmeth.2022.100203 PMC9046455

[59] H. M. Dalton, S. P. Curran, Hypodermal responses to protein synthesis inhibition induce systemic developmental arrest and AMPK-dependent survival in Caenorhabditis elegans. PLOS Genet. 14, e1007520 (2018). 30020921 10.1371/journal.pgen.1007520 PMC6066256

[60] N. Zuela, J. Dorfman, Y. Gruenbaum, Global transcriptional changes caused by an EDMD mutation correlate to tissue specific disease phenotypes in *C. elegans*. Nucleus 8, 60–69 (2017). 27673727 10.1080/19491034.2016.1238999 PMC5287205

[61] A. Brull, B. Morales Rodriguez, G. Bonne, A. Muchir, A. T. Bertrand, The pathogenesis and therapies of striated muscle laminopathies. Front. Physiol. 9, 1533 (2018). 30425656 10.3389/fphys.2018.01533 PMC6218675

[62] F. J. Ramos, S. C. Chen, M. G. Garelick, D.-F. Dai, C.-Y. Liao, K. H. Schreiber, V. L. MacKay, E. H. An, R. Strong, W. C. Ladiges, P. S. Rabinovitch, M. Kaeberlein, B. K. Kennedy, Rapamycin reverses elevated mTORC1 signaling in Lamin A/C–deficient mice, rescues cardiac and skeletal muscle function, and extends survival. Sci. Transl. Med. 4, 144ra103 (2012). 10.1126/scitranslmed.3003802 PMC3613228 22837538

[63] C. Mayer, I. Grummt, Ribosome biogenesis and cell growth: mTOR coordinates transcription by all three classes of nuclear RNA polymerases. Oncogene 25, 6384–6391 (2006). 17041624 10.1038/sj.onc.1209883

[64] G. Pietrafesa, R. De Zio, S. I. Scorza, M. F. Armentano, M. Pepe, C. Forleo, G. Procino, A. Gerbino, M. Svelto, M. Carmosino, Targeting unfolded protein response reverts ER stress and ER Ca^2+^ homeostasis in cardiomyocytes expressing the pathogenic variant of Lamin A/C R321X. J. Transl. Med. 21, 340 (2023). 37217929 10.1186/s12967-023-04170-y PMC10204200

[65] J. B. DuRose, D. Scheuner, R. J. Kaufman, L. I. Rothblum, M. Niwa, Phosphorylation of eukaryotic translation initiation factor 2α coordinates rRNA transcription and translation inhibition during endoplasmic reticulum stress. Mol. Cell. Biol. 29, 4295–4307 (2009). 19470760 10.1128/MCB.00260-09 PMC2715810

[66] A. Okamoto, M. Koike, K. Yasuda, A. Kakizuka, Maintaining ATP levels via the suppression of PERK-mediated rRNA synthesis at ER stress. Biochem. Biophys. Res. Commun. 394, 42–47 (2010). 20171949 10.1016/j.bbrc.2010.02.065

[67] J. Odell, Y. Tang, Y. S. Ambekar, G. R. Kidiyoor, H. Saadi, G. F. Woodworth, L. J. Holt, G. Scarcelli, H. Yu, J. Lammerding, The Deformability of the Mammalian Cell Nucleus is Determined by the Identity of the Lamin Rod Domain. bioRxiv 2025.07.22.666133 [Preprint] (2025). 10.1101/2025.07.22.666133.

[68] D. Valverde-Mendez, A. M. Sunol, B. P. Bratton, M. Delarue, J. L. Hofmann, J. P. Sheehan, Z. Gitai, L. J. Holt, J. W. Shaevitz, R. N. Zia, Macromolecular interactions and geometrical confinement determine the 3D diffusion of ribosome-sized particles in live *Escherichia coli* cells. Proc. Natl. Acad. Sci. U.S.A. 122, e2406340121 (2025). 39854229 10.1073/pnas.2406340121 PMC11789073

[69] S. Brenner, The genetics of Caenorhabditis elegans. Genetics 77, 71–94 (1974). 4366476 10.1093/genetics/77.1.71 PMC1213120

[70] R. Kamath, Genome-wide RNAi screening in Caenorhabditis elegans. Methods 30, 313–321 (2003). 12828945 10.1016/s1046-2023(03)00050-1

[71] R. S. Kamath, M. Martinez-Campos, P. Zipperlen, A. G. Fraser, J. Ahringer, Effectiveness of specific RNA-mediated interference through ingested double-stranded RNA in Caenorhabditis elegans. Genome Biol. 2, RESEARCH0002 (2000). 11178279 10.1186/gb-2000-2-1-research0002 PMC17598

[72] A. K. Allen, J. E. Nesmith, A. Golden, An RNAi-based suppressor screen identifies interactors of the Myt1 ortholog of *Caenorhabditis elegans*. G3 4, 2329–2343 (2014). 25298536 10.1534/g3.114.013649 PMC4267929

[73] N. Otsu, A threshold selection method from gray-level histograms. IEEE Trans. Syst. Man Cybern. 9, 62–66 (1979).

