# Supplementary material for: Muscular dystrophy–associated lamin variants disrupt cellular organization through a nucleolar-ribosomal axis
Source: Sci Adv. 2026 Aug 26;12(35):eaec9028. doi: 10.1126/sciadv.aec9028 (PMC13510739; doi:10.1126/sciadv.aec9028)
Supplement: Supplementary file 1 [file sciadv.aec9028_sm.pdf]

Science Advances
AAAS

Supplementary Materials for

Muscular dystrophy–associated lamin variants disrupt cellular organization through a nucleolar-ribosomal axis

Xiangyi Ding *et al.*

Corresponding author: Daniel A. Starr, dastarr@ucdavis.edu; G. W. Gant Luxton, ggluxton@ucdavis.edu

Sci. Adv. **12**, eaec9028 (2026)
DOI: 10.1126/sciadv.aec9028

The PDF file includes:

Table S1
Figs. S1 to S5
Legends for movies S1 and S2

Other Supplementary Material for this manuscript includes the following:

Movies S1 and S2

Table S1. Strain list

Strain Name	Genotype	References
**UD803**	*ycSi4[pSL848; P_{ges}-1::40nmGEM::eGFP::unc-54 3'UTR] IV*	(*12*)
**UD838**	*ycSi5[pSL850; P_{semo}-1::40nmGEM::eGFP::unc-54 3'UTR] IV*	(*12*)
**UD1055**	*lmn-1(yc96[Y59C]) I/hT2[bli-4(e937) let-?(q782) qIs48] (I;III); ycSi4 IV*	*This study*
**UD1054**	*lmn-1(yc96[Y59C]) I/hT2 (I;III); ycSi5 IV*	*This study*
**UD1043**	*lmn-1(yc105[R64P]) I/hT2[bli-4(e937) let-?(q782) qIs48] (I;III); ycSi4 IV*	*This study*
**UD916**	*lmn-1(yc105[R64P]) I/hT2 (I;III); ycSi5 IV*	*This study*
**UD1042**	*lmn-1(yc105[R64P]) I/hT2 (I,III); ycSi2 [pSL845 P_{semo-1}::mKate2::tram-1] IV*	*This study*
**COP262**	*unc-119(ed3) III; knuSi221 [P_{fib-1}::fib-1::eGFP::fib-1 3'UTR+unc-119(+)] II*	(*72*)
**UD1079**	*knuSi221 II; lmn-1(yc105[R64P]) I/hT2 (I;III); ycSi2 IV*	*This study*
**UD863**	*yc113 [GFP_{11}::rps-18] IV; ycSi2 IV; juEx5375 [P_{col-19}::GFP_{1-10}+ P_{ttx-3}::RFP]*	(*12*)
**UD1079**	*yc113 IV; juEx5375; lmn-1(yc105[R64P]) I/hT2 (I;III)*	*This study*
**UD1180**	*yc113 IV; juEx5375; lmn-1(yc96[Y59C]) I/hT2 (I;III)*	*This study*
**UD883**	*lmn-1 (yc96[Y59C]) I/hT2 (I;III); ycIs10[P_{col-10}::nls::gfp::lacZ] V, him-8(e1489) IV*	(*17*)
**UD895**	*lmn-1 (yc105[R64P]) I/hT2 (I;III); ycIs10 V, him-8(e1489) IV*	(*17*)
**UD1041**	*emr-1(gk119) I; ycSi4 IV*	*This study*
**UD1044**	*emr-1(gk119) I; ycSi5 IV*	*This study*
**UD1046**	*lem-2(tm1582) II; ycSi4 IV*	*This study*
**UD1045**	*lem-2(tm1582) II; ycSi5 IV*	*This study*
**UD977**	*unc-84(n369) X; ycSi4 IV*	*This study*
**UD875**	*unc-84(n369) X; ycSi5 IV*	*This study*
**UD1018**	*unc-83(e1408) V; ycSi4 IV*	*This study*
**UD1019**	*unc-83(e1408) V; ycSi5 IV*	*This study*
**DUP311**	*garr-1(sam179[garr-1::wrmScarlet(1-11)]) ;nucl-1(sam186[nucl-1::sGFP(1-11)])IV*	*(32)*
**CZ18550**	*juSi123 [rpl-29::GFP] II; rpl-29(tm3555) IV*	*(33)*
**UD1177**	*juSi123 [rpl-29::GFP] II; lmn-1(yc105[R64P]) I/hT2 (I;III)*	*This study*
**UD1178**	*juSi123 [rpl-29::GFP] II; lmn-1(yc96[Y59C]) I/hT2 (I;III)*	*This study*

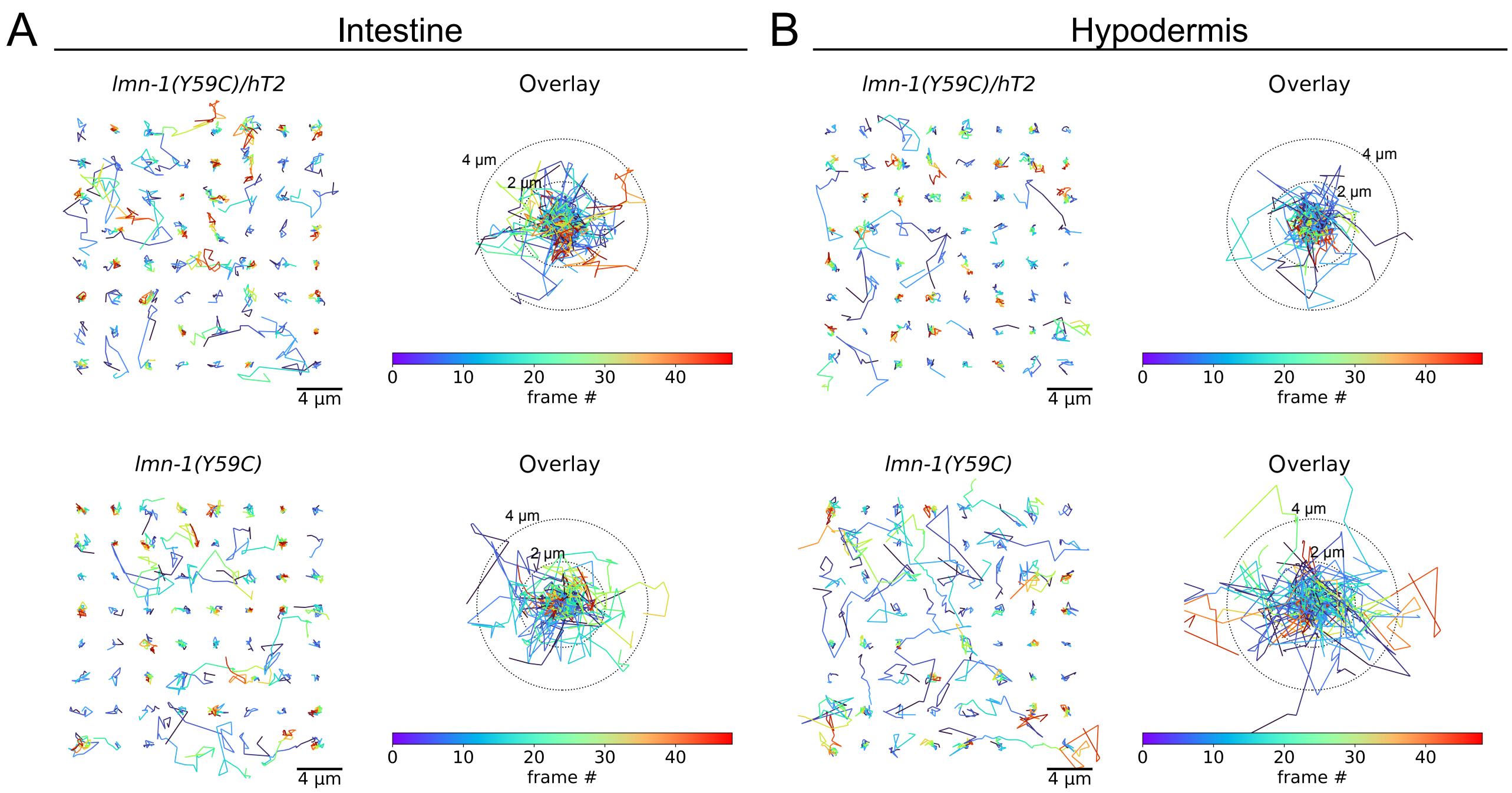

Figure S1.Homozygous *lmn-1(Y59C)* mutants exhibit increased GEM mobility in the hypodermis but show minimal changes in the intestine. Representative GEM trajectories color-coded by frame number are shown for (A) intestine and (B) hypodermis of the indicated strains. Scale bar: 4 µm. Concentric circles denote 2 µm and 4 µm radii from the trajectory origin.

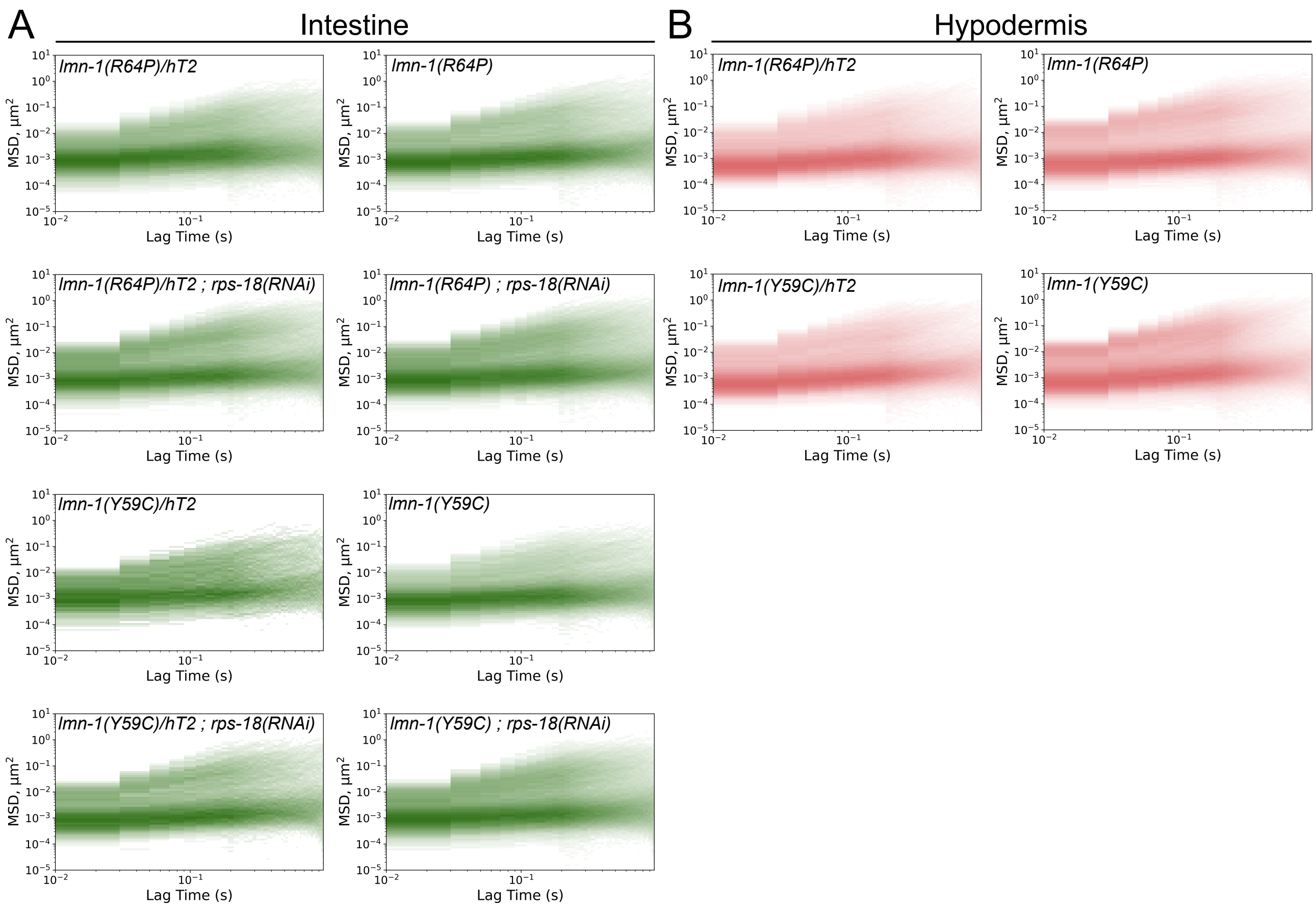

Figure S2. Mean squared displacement (MSD) analysis of each GEM mobility shows minimal population shifts in *lmn-1* mutants. Mean squared displacement (MSD) analysis of GEM mobility over time displayed on a log-log scale for intestinal (A) and hypodermal (B) tissues. Data shown for *lmn-1(R64P)/hT2* heterozygotes, *lmn-1(R64P)* homozygotes, *lmn-1(Y59C)/hT2* heterozygotes, *lmn-1(Y59C)* homozygotes, and corresponding intestinal GEM strains treated with *rps-18(RNAi)*. Each plot displays MSD (μm²) versus lag time (s) with color intensity representing track density. Unlike the population shifts observed with *anc-1(e1873)*(12), these *lmn-1* mutants show relatively subtle changes in the distribution between constrained and unconstrained GEM populations. The overall bimodal pattern is preserved across all conditions, with dense clustering at lower MSD values (constrained population) and more dispersed patterns at higher MSD values (unconstrained population).

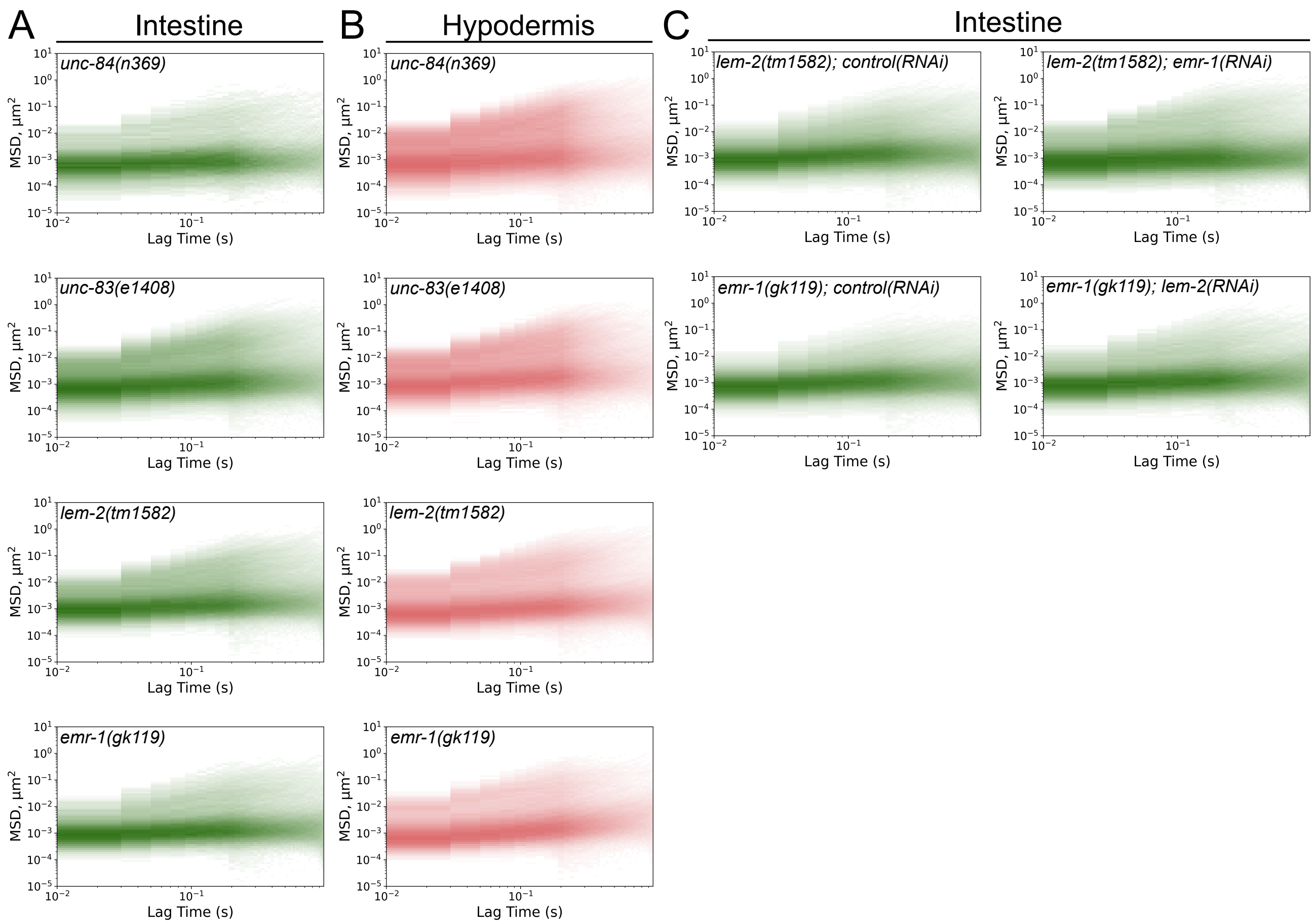

Figure S3. Mean squared displacement (MSD) analysis of each GEM mobility shows minimal population shifts in nuclear envelope protein mutants. Mean squared displacement (MSD) analysis of GEM mobility over time displayed on a log-log scale for intestinal (A, C) and hypodermal (B) tissues. A-B) Data shown for *unc-84(n369)*, *unc-83(e1408)*, *lem-2(tm1582)*, and *emr-1(gk119)* single mutants. C) Data shown for *lem-2(tm1582); control(RNAi), lem-2(tm1582); emr-1(RNAi), emr-1(gk119); control(RNAi),* and *emr-1(gk119); lem-2(RNAi)* conditions. Each plot displays MSD (µm²) versus lag time (s) with color intensity representing track density. The overall bimodal pattern is preserved across all conditions, with dense clustering at lower MSD values (constrained population) and more dispersed patterns at higher MSD values (unconstrained population).

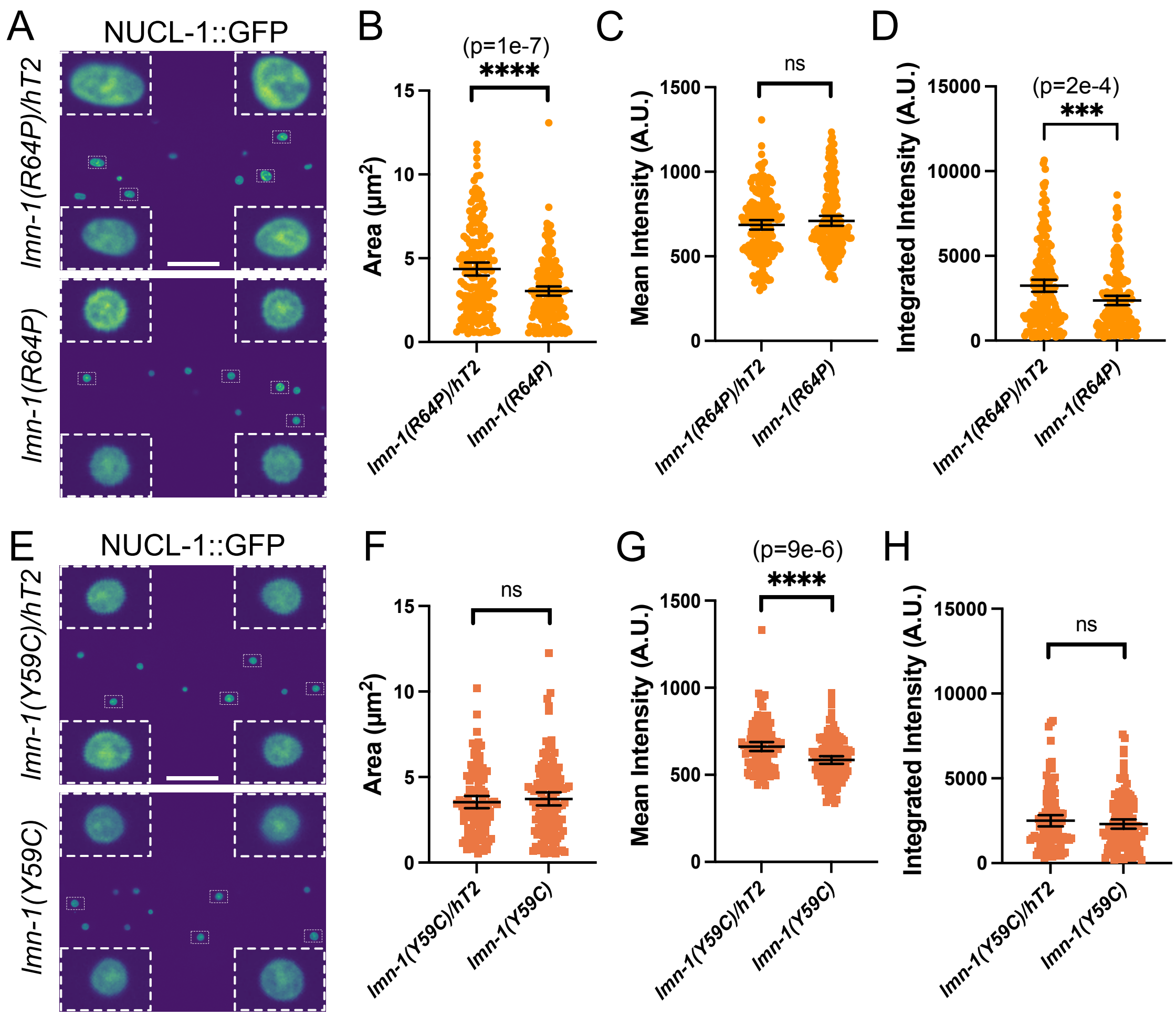

Figure S4. NUCL-1::GFP reveals additional nucleolar alterations in *lmn-1* mutants. A and E) Representative spinning-disk confocal images of NUCL-1::GFP in the hypodermis of day 1 adult *lmn-1(R64P)/hT2* and *lmn-1(R64P)* animals (A), and *lmn-1(Y59C)/hT2* and *lmn-1(Y59C)* animals (E). Insets show magnified views of representative nucleoli. Fluorescence is displayed using the viridis colormap. Scale bar, 10 µm. B-D, F-H) Quantification of nucleolar area (B, F), mean NUCL-1::GFP intensity (C, G), and integrated NUCL-1::GFP intensity (D, H) in the indicated genotypes. Each dot represents one nucleolus. (B-D: n = 181 and 177 nucleoli from 13 worms per genotype; F-H: n = 113 and 127 nucleoli from 11 and 13 worms per genotype, left to right, respectively); bars indicate mean ± 95% CI. Statistical significance was assessed by two-tailed Welch's t test, with exact p values shown above each comparison. Asterisks denote significance as follows: ****, P < 0.0001; ***, P ≤ 0.001; **, P ≤ 0.01; *, P ≤ 0.05; ns, P > 0.05.

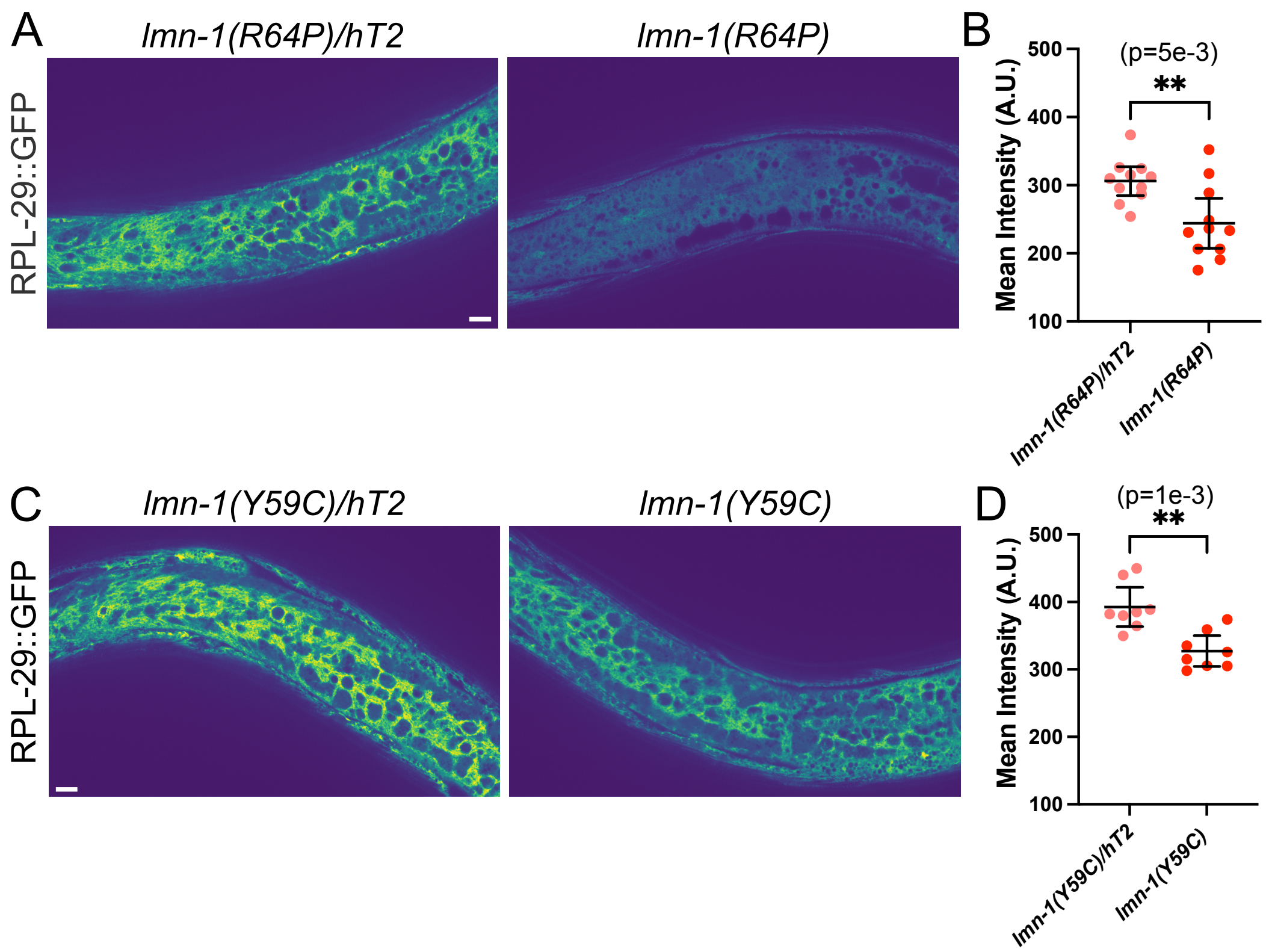

Figure S5. RPL-29::GFP reveals reduced ribosome abundance in *lmn-1* mutants. A, C) Representative spinning-disk confocal images of RPL-29::GFP fluorescence in the hypodermis of day 1 adult *lmn-1(R64P)/hT2* and *lmn-1(R64P)* animals (A), and *lmn-1(Y59C)/hT2* and *lmn-1(Y59C)* animals (C). Fluorescence intensity is displayed using the viridis colormap. Scale bar: 10 µm. B,D) Quantification of mean RPL-29::GFP fluorescence intensity in the hypodermis of the indicated genotypes. Each dot represents one worm. Bars indicate mean ± 95% CI. Statistical significance was assessed by two-tailed Welch's t test; exact p values shown above each comparison. Asterisks denote significance as follows: ****, $P < 0.0001$; ***, $P \leq 0.001$; **, $P \leq 0.01$; *, $P \leq 0.05$; ns, $P > 0.05$.

Movie S1. Representative movie of a *C. elegans* day 1 young adult carrying the transgene *ycSi4[pSL848; pges-1::40nmGEM::eGFP::unc-54 3'UTR] IV*, showing GEMs movement in the intestine. GEMs are pseudocolored in green. *lmn-1(R64P)/hT2* (left) and *lmn-1(R64P)* (right) mutant worms are shown side-by-side. Time bar and scale bar are indicated.

Movie S2. Representative movie of a *C. elegans* day 1 young adult carrying the transgene *ycSi5[pSL850; psemo-1::40nmGEM::eGFP::unc-54 3'UTR] IV*, showing GEMs movement in the hypodermis. GEMs are pseudocolored in green. *lmn-1(R64P)/hT2* (left) and *lmn-1(R64P)* (right) mutant worms are shown side-by-side. Time bar and scale bar are indicated.
